# Intuitive decision making as a gradual process: investigating semantic intuition‐based and priming‐based decisions with fMRI

**DOI:** 10.1002/brb3.420

**Published:** 2015-12-22

**Authors:** Thea Zander, Ninja K. Horr, Annette Bolte, Kirsten G. Volz

**Affiliations:** ^1^ Werner Reichardt Centre for Integrative NeuroscienceUniversity of TübingenTübingenGermany; ^2^ International Max Planck Research SchoolTübingenGermany; ^3^ Research Centre for Computational Neuroscience and Cognitive RoboticsUniversity of BirminghamBirminghamUK; ^4^ Technische Universität DresdenDresdenGermany; ^5^ Department of PsychologyUniversity of BaselSwitzerland

**Keywords:** Conceptual priming, intuitive decision making, neural activity suppression, orbitofrontal cortex, two‐stage model of intuition

## Abstract

**Introduction:**

Intuition has been defined as the instantaneous, experience‐based impression of coherence elicited by cues in the environment. In a context of discovery, intuitive decision‐making processes can be conceptualized as occurring within two stages, the first of which comprises an implicit perception of coherence that is not (yet) verbalizable. Through a process of spreading activation, this initially non‐conscious perception gradually crosses over a threshold of awareness and thereby becomes explicable. Because of its experiential basis, intuition shares conceptual similarities with implicit memory processes. Based on these, the study addresses two research questions: (1) Is the gradual nature of intuitive processes reflected on a neural level? (2) Do intuition‐based decisions differ neurally from priming‐based decisions?

**Methods:**

To answer these questions, we conducted an fMRI study using the triads task and presented participants with coherent word triads that converge on a common fourth concept, and incoherent word triads that do not converge on a common fourth concept. Participants had to perform semantic coherence judgments as well as to indicate whether they immediately knew the fourth concept. To enable investigating intuition‐based and priming‐based decisions within the same task and with the same participants, we implemented a conceptual priming procedure into the coherence judgment task. We realized this by priming participants with concepts associated with incoherent triads in separate priming blocks prior to the coherence judgments.

**Results:**

For intuition‐based decisions, imaging results mainly revealed activity within the orbitofrontal cortex, within the inferior frontal gyrus and the middle temporal gyrus. Activity suppression in the right temporo‐occipital complex was observed for priming‐based decisions.

**Conclusions:**

With respect to research question 1, our data support a continuity model of intuition because the two intuitive stages show quantitatively distinct brain activation patterns. Regarding research question 2, we can draw the preliminary conclusion of a qualitative difference between intuition‐based and priming‐based decisions.

## Introduction

### A two‐stage model of intuition

People have to make decisions every day. Often, they have to make them quickly, without the information they might need to fully understand a situation or to foresee the possible consequences of the choices they make. Decisions like these, where one does not go through all possible alternatives and steps of reasoning beforehand, are called intuitive (e.g., Claxton 1998; Sinclair and Ashkanasy 2005; Bolte and Goschke 2008; Evans 2008; Gigerenzer 2008; Sadler‐Smith 2008; Betsch and Glöckner 2010; Epstein 2010; Hogarth 2010; Myers 2010; Volz and Zander 2014). Research on intuitive processing is still a young endeavour seeking for conceptual clarification as well as an anatomical mapping of intuitive functioning. The present functional magnetic resonance imaging (fMRI) study set out to contribute to the ongoing debate on the topic providing behavioral and neural results that may help to understand the concept better.

Within a context of discovery, Bowers et al. (1990) have put forward the idea that intuitive decision‐making is the immediate perception of coherence in the environment. According to these authors, intuitive decision‐making can be conceived of as “a preliminary perception of coherence (pattern, meaning structure) that is at first not consciously represented, but which nevertheless guides thought and inquiry towards a hunch or hypothesis about the nature of the coherence in question” (p. 74). They conceptualized the intuitive decision‐making process in a two‐stage model: In the first stage, through a process of automatically spreading activation that is evoked by certain clues of coherence in the sensory input, the decision maker has a “tacit or implicit perception of coherence” (p. 74). She is however not yet able to explicitly verbalize the source of this impression. In this first stage, called *guiding stage* of intuition, “clues to coherence activate relevant mnemonic and semantic networks in a graded and cumulative fashion” (p. 74), giving rise to a preliminary intuitive feeling. The authors postulate that “the tacit perception of coherence guide[…] people gradually to an explicit representation of it in the form of a hunch or hypothesis” (p. 72). They further elaborate that “[e]ventually the level of patterned activation is sufficient to cross a threshold of consciousness, and at that point, it is represented as a hunch or hypothesis” (p. 72). In this overcoming the threshold of awareness we then see the second, *integrative stage* of intuition.^1^

### The gradual unfolding of intuitive processing

Within this two‐stage model, Bowers et al. (1990) propose that the cognitive processes that underlie intuitive hunches are continuous rather than discontinuous in nature. According to this continuity model, intuition is conceived of as a *gradual process* leading from the first immediate implicit perception of a complex and vague input to a more explicit experience characterized by being able to verbalize why and how certain pieces of (semantic) information might belong together. Thus the impression of coherence builds up implicitly over time. The more environmental cues hint in one particular direction, in that way accruing meaning, the more representations are activated in memory. This model may be related to the idea that unconscious thought organizes information. For instance, Ritter and Dijskterhuis (2014), recently proposed, based on their empirical findings, that representations become better organized and more polarized, and that memory becomes more gist‐based, during an unconscious thought period (i.e., in an incubation period). Their results may suggest that unconscious thought is a process wherein disorganized information becomes more and more organized until some kind of threshold is reached and conclusions can be transferred to consciousness.

To empirically test their two‐stage conceptualization of intuition, Bowers et al. (1990) developed several experimental paradigms, one of which, the triads task, is now widely used to investigate intuitive decision processes (Bolte and Goschke 2005; Ilg et al. 2007; Topolinski and Strack 2009a,b; Remmers et al. 2014). In this task, participants are asked to assess the semantic coherence of word triads (i.e., three words presented below each other that are either semantically linked through the existence of a forth word that describes this link or are semantically unrelated), which has been called semantic coherence judgment. Participants are instructed not only to indicate whether they think the triad is semantically coherent but also to make an attempt to find the solution, that is, to explicitly name the common associate (CA) of the three words; both the coherence judgment and the attempt to name the CA require the activation of distantly related concepts in semantic memory. Therewith it is possible to determine in which intuitive stage a person is: If the participant judges a word triad correctly as coherent, but cannot name a possible solution, this is an indicator of her being in the guiding stage of intuition. If the participant, however, judges a triad correctly as coherent and is additionally able to name a correct CA, this is an indicator of her being in the integrative stage of intuition (Bowers et al. 1990). The typical empirical finding is that participants are remarkably correct in discriminating between coherent and incoherent triads, even in the guiding stage, that is, when they are not able to explicitly name the CA (Bowers et al. 1990; Bolte et al. 2003; Bolte and Goschke 2005; Ilg et al. 2007; Topolinski and Strack 2008; Remmers et al. 2014). In fact, results have been interpreted in favour of a genuine continuity in the underlying perceptual‐cognitive processing of information based on the phenomenon that semantic processing evoked by the sensory input spreads out steadily and in that way may converge on common semantic nodes (i.e., the solution concepts, CAs). Consequently, the automatically spreading activation is assumed only for coherent triads to converge on a CA, for incoherent triads the semantic spread is assumed to fizzle out; the linchpin of each empirical investigation using the triads task is thus to assure that the material used works that way (Collins and Loftus 1975; Anderson 1983; Bowers et al. 1990). Besides the behavioral results by Bowers et al. (1990), similarly, a recent magnetencephalography (MEG) study in the visual domain (Horr et al. 2014) also gives evidence in favor of this continuity model, in that activation reflecting non‐conscious processes in the guiding stage of intuition showed very similar patterns as activation reflecting conscious processes in the integrative stage. Thus, these data show a quantitative rather than a qualitative difference in the two processing stages.

In contrast to a continuity model, a discontinuous conceptualization of intuition would assume something like an “Aha!’ moment” that indicates an all‐of‐a‐sudden insight into the triad's solution. The underlying process here is assumed to be an extensive mental restructuring of the problem space that finally, after a momentary standstill (which has been termed *impasse* in the insight literature), leads to a new way of thinking and thus to a sudden insight (cf. Ohlsson 1992; Knoblich and Öllinger 2006). According to this view, the transition from the guiding to the integrative stage of intuition will be experienced as a sudden perception of coherence or insight that seems virtually self‐validating.

Which model best describes the underlying cognitive and neural processes taking place in the triads task remains an open research question – that we addressed in this fMRI study – since a gradual process has been indirectly assumed but not directly empirically tested on a neural level yet. That is, according to a *continuity model*, coherence judgments proceed in a graded fashion from problem formulation to problem solution as previously encoded information is activated by clues to coherence. Therewith the continuous build‐up of coherent information is considered to be in itself sufficient to cross a threshold of awareness. In a *discontinuity model*, however, intuitive coherence judgments indicate a discontinuous increase in associative proximity of responses to the solution concept as the decision maker proceeds from early to later clues – therewith reflecting a more or less spontaneous restructuring of the problem that immediately yields its solution. While there is some empirical evidence to support a continuity model of intuition (Bowers et al. 1990; Horr et al. 2014), the present study is the first to directly compare the models, not only on a behavioral level, but on a neural one as well. Based on previous evidence, therefore, our first hypothesis is: The intuitive perception of semantic coherence builds up gradually over time, and therefore a continuity model fits better to explain behavioral performance and neural activation in the triads task.

### Intuition and priming

Our second research question focusses on the underlying processes of intuitive decision making in relation to implicit memory processes as follows: The definition of intuition that Bowers et al. 1990) put forward (as outlined above), seizes on (1) the aspect of rapidity in intuitive judgments, (2) the lack of an explicit basis for decisions made intuitively and (3) the stimulative nature of intuition to initiate and guide decisions. Hence it concurs with other definitions of intuition as a (1) quick, and (2) mostly non‐conscious process (i.e., with regard to the underlying cognitive processes as well as the source of the decision), which is (3) based on tacit knowledge and (4) results in some sort of feeling gravitating towards an idea or hunch that is strong enough to act upon (e.g., Betsch 2008; Sadler‐Smith 2008; Glöckner and Witteman 2010; Hogarth 2010; Gigerenzer and Gaissmaier 2011; Volz and Zander 2014). It is interesting that this apprehension of intuition seems to coincide with what has been conceived of as implicit memory processes. As discussed in recent contributions (see Volz 2012; Volz and Zander 2014), one could ask whether, given the conceptualization of intuition as the ability to create an idea or solution (mostly on the basis of implicitly acquired knowledge) even if one cannot explain how one arrived at it, intuitive decision processes and implicit memory mechanisms are simply two sides of the same coin. According to Schacter (1992), implicit memory has been defined as “an unintentional, non‐conscious form of retention that can be contrasted with explicit memory, which involves conscious recollection of previous experiences” (p. 559). Thus, when defining implicit memory, researchers primarily emphasize its involuntary and non‐conscious character; implicitly acquiring knowledge is something that runs on the sideline without any reference to encoding and storage (Tulving et al. 1982; Schacter 1987, 1992; Roediger 1990; Tulving and Schacter 1990; Schacter et al. 1993; Schott et al. 2002; Henson 2003; Richardson‐Klavehn 2010; Goschke and Bolte 2012). This phenomenology resembles the *feeling of knowing something without remembering why or wherefrom it is known*, which occurs in intuitive processing (cf. Claxton 1998). Building on our recent synopsis (Volz and Zander 2014), which suggests that the two concepts “differ substantially both in the format in which information is assumed to be stored in memory and used for a decision, as well as in the kind of signal accompanying the respective cognitive processes” (p. 32ff), in this fMRI study we directly test this assumption on the neural level. Our second hypothesis is therefore: Intuitive and implicit memory processes are not alike with regard to their neural correlates and differ qualitatively. Here we use priming as a test case of one form of implicit memory, since it is the most suitable one on which to base a neural comparison (cf. Volz and Zander 2014).

### Study overview, aim, and hypotheses

Neural imagining is an ideal method for addressing both of our research questions since it is suited to assessing similarities and differences on a cognitive level and can at the same time give evidence as to whether those differences are qualitative or quantitative. Using a modified version of Bowers et al. (1990) triads task and implementing a conceptual priming procedure into it, we investigated priming‐based and intuitive decision processes within the same task and with the same participants.

In terms of hypothesis 1, we expected on the behavioral level to replicate previous findings (e.g. that people are able to discriminate between coherent and incoherent triads above chance level, even if they cannot come up with a solution concept). For hypothesis 2, we expected to find that priming‐based decisions are made more quickly than non‐primed decisions, which would indicate a successful priming procedure. We further assumed that participants would indicate primed trials as coherent due to a process of misattributing semantic meaning elicited by the previous priming to objectively incoherent triads (for a description of the conceptual priming process, see the Methods section).

On the neuronal level, for hypothesis 1, we expected the activation patterns to support a continuity model of intuition. Specifically, we assumed that we would find a gradual increase of activation within a network comprising the orbitofrontal cortex (OFC), the left inferior frontal gyrus (IFG, BA 47), the superior temporal sulcus (STS), and the anterior insula. In a (preliminary) neuro‐cognitive model of intuition, the OFC has been suggested to serve as a rapid detector and predictor of potential content by sending this initial signal to down‐stream areas (Bar et al. 2006; Volz and von Cramon 2006; Volz et al. 2008; Luu et al. 2010; Horr et al. 2014). Activation within the IFG is expected to occur for intuitive semantic coherence judgments as this area is known to be specifically involved in the processing of semantic relationships between words and/or phrases as well as in the retrieval of semantic information (Bookheimer 2002). We further expected to find activation within the STS, since a recent study on intuitive semantic coherence judgments, which also used the triads task as we did, but did not test for potential OFC activation, suggested that this area reflects the integration of remote semantic associations (Ilg et al. 2007). We also expected activation within the anterior insula, which has been shown to correlate with levels of non‐conscious interoceptive awareness, that is, the ability to perceive afferent information, an ability believed to contribute to the intuitive apprehension of meaning (Critchley et al. 2004; Craig 2009). According to Bowers et al. (1990) conceptualization, the activation pattern reflecting intuitive decision processes should expand from a guiding stage to an integrative stage. Thus we expected the activation pattern for semantic coherence judgments to be stronger in cases where a person has already crossed the threshold of awareness and is in the integrative stage of intuition, that is, when the person explicitly knows the CA. If, however, contrary to our expectations, a qualitative rather than quantitative difference distinguishes non‐conscious processes of the guiding stage of intuition from the more explicit processes of the integrative stage and therefore qualitatively distinct activation patterns are found in each of the two stages, the data would support a discontinuity model of intuition.

In terms of hypothesis 2, we expected intuition‐based and priming‐based coherence judgments to differ qualitatively with regard to their neural correlates. Specifically, we expected that priming‐based decisions would not draw on the network of areas suggested to support intuitive decision processes (see above), but rather would be characterized by a deactivation within inferior frontal and/or temporo‐occipital areas (Demb et al. 1995; Schacter et al. 1998; Wagner et al. 2000; Henson 2003). The literature on priming reveals that both perceptual and conceptual priming result in a domain‐specific deactivation in the computing areas. This has been interpreted as signal reduction, wherein, it is postulated, less energy is needed when a person encounters the same or a related stimulus twice (e.g., Martin et al. 1995; Blaxton et al. 1996; Buckner et al. 1998). Moreover, areas within the inferior prefrontal cortex and the right occipital cortex as well as fusiform areas have been suggested to specifically reflect initial semantic processing (Squire et al. 1992; Demb et al. 1995).

Whether there are overlapping patterns of activation for intuition‐based and priming‐based decisions would thus be tested by investigating both deactivation for intuitive decision‐making processes and OFC activation for priming processes, a procedure which, to our knowledge, has not been used in this context before.

Furthermore, in order to guarantee for our stimulus material that only in case of coherent triads the semantic spread of activation automatically converges on a CA, we first run a behavioral pre‐study. We expected coherent triads to produce faster response times to their CAs than to unrelated or non‐words directly after lexical decisions. For incoherent triads, we did not expect a difference in response times to unrelated or non‐words.

## Methods

### Participants

Twenty‐five students (16 female, mean age 25.8 years, SD 4.37, range 20–37) from the University of Tübingen took part in the fMRI experiment. They were all right‐handed and healthy (i.e., without any reported history of neurological or psychiatric illness), and spoke German as their native language. Handedness was assessed with the Edinburgh Handedness Inventory (Oldfield 1971). Active vocabulary was assessed using a German vocabulary test called WST (Schmidt and Metzler 1992). Participants showed an average performance of 35.92 (SD = 1.8) of correct responses, which is a typical result for young adults with a qualification for university entrance. Since we used a quasi‐balanced design with regard to “gender”, we ran an analysis in which the means of the intuition indices were compared for men (*m* = 14.13, SE = 3.8) and women (*m* = 15.15, SE = 4.6). No statistical differences were found (*t*(17) = −0.150, *P *=* *0.882). Hence, any gender differences could be ruled out. All participants gave written informed consent before being tested and were paid 12 Euros per hour for their attendance. Four participants had to be excluded due to technical problems. Further two participants were excluded as they indicated in the feedback questionnaire that they had had severe problems in performing the task and, in fact, their results showed a deviant response pattern (both indicated more than 80% of the triads to be incoherent and did not provide any potential CA in the post‐scan questionnaire). In the end, the data from 19 participants was analyzed for the present study and is reported below. The experimental procedure and data collection/storage followed the ethical guidelines of the “Declaration of Helsinki” (revised version, 2012) and was reviewed and approved by the local ethical committee of the University Hospital of Tübingen and the Medical Department.

### Behavioral pre‐study: assessing the automatic spread of activation in the stimulus material

In the triads task, based on the theory, semantic processing for coherent and incoherent triads is assumed to differ in that semantic activation initiated by the three clue words of a triad spreads out automatically and converges on a common concept. This, however, is only the case for coherent triads; in the case of incoherent triads the semantic spread fizzles out. Since this theorizing of intuitive judgments in the context of discovery crucially hinges on the assumption that only in the case of coherent triads is the intuitive impression elicited by a process of automatically spreading activation (ASA), we tested that assumption directly in our stimulus material. As mentioned in the introduction, the ASA is considered a very fast phenomenon (i.e., in terms of a rapidly occurring phenomenon), occurring directly after semantic information is presented in order to internally prime the concept that all the distinct pieces of semantic information have in common. So in a behavioral pre‐study we presented 25 participants (19 female, mean age 24.04, SD 2.73, range 20–30) – other than that of the fMRI study – with a triad (coherent or incoherent) for 1.3 sec and instructed them to simply read the three words. After the presentation of the triad, they had 2 sec to perform a lexical decision task (word versus non‐word decision). Thus participants in this behavioral pre‐study did *not* have to judge the semantic coherence of the triad at any time but simply made lexical decisions after having read the word triads. To capture the exact moment when the ASA occurred, we investigated two different time points: The lexical decision task was presented either at 20 msec (t1) or 1200 msec (t2) after participants had read the word triads.

**Figure 1:**
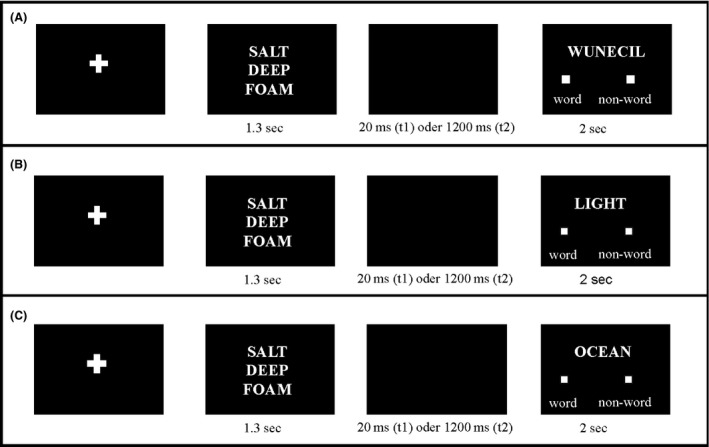
Experimental design behavioral pre‐study. (A) depicts a coherent triad, followed by a non‐word in the lexical decision task. (B) depicts a coherent triad, followed by a semantically unrelated word, and (C) depicts a coherent triad, followed by the actual (i.e., preordained) solution (i.e., the CA). Incoherent triads were only used as controls and could be either followed by a non‐word or by a word semantically unrelated to all its constituents. Participants were not informed about the existence of the two different triad types (coherent/incoherent); they were just instructed to read the three words and to perform the lexical decision task. To ensure that participants indeed read the three words in the beginning of each trial, they were told that we would re‐present them with some of the words after the experiment and that they had to discriminate then between old and new words.

For coherent triads (e.g., SALT, DEEP, FOAM), words in the lexical decision task were either the actual CAs of the triad (i.e., SEA), or semantically unrelated words (e.g., DESK) or non‐words (e.g., WUNECIL). For incoherent triads (e.g., CADET, CAPSULE, BOAT), words in the lexical decision task were either semantically unrelated words (e.g., BOTTLE) or non‐words (e.g., RABIHAL). For a detailed description of the design and trials of this behavioral pre‐study see Figure 1. We assumed that reaction times (RTs) in the lexical decision task would be faster when the participant encountered a corresponding solution concept (i.e., the CA), which would indicate that the ASA only becomes activated in the event of a coherent triad, where the three constituents internally prime the common concept of the three words. In accordance with the results of Bolte and Goschke (2005), who found out that participants were able to perform intuitive coherence judgments very quickly (within a time window of 1.5 sec), we expected t1 to be the critical time where we might prove the ASA and expected that RTs of corresponding CAs for coherent triads were faster only at t1, not at t2. The RT results did in fact show exactly this pattern, which can be seen in Table 1: Interestingly, only at the early time point (i.e., at t1) did the three clue words internally prime the solution concept, which is revealed by significantly faster RTs occurring exclusively in response to real solution concepts having been displayed in the lexical decision task (*F*(1, 29) = 30.28, *P *<* *0.01). This was not the case with the later point in time (i.e., t2), where having faced a coherent triad did not give any advantage in responding to the corresponding solution concept. We interpret our results as demonstrating that the ASA can be elicited specifically by our stimulus material. Concretely, we were able to show that the ASA is specific to the processing of coherent word triads, thereby confirming the hypothesis that this holds true only at an early time point.

**Table 1:** Lexical decision reaction times of the behavioral pre‐study in milliseconds with standard deviations in parentheses

	Actual solutions	Unrelated words
T1	758.83 (± 124.15)	800.73 (± 167.67)
T2	812.38 (± 143.65)	796.40 (± 184.80)

### Task and paradigm of the fMRI study

**Figure 2:**
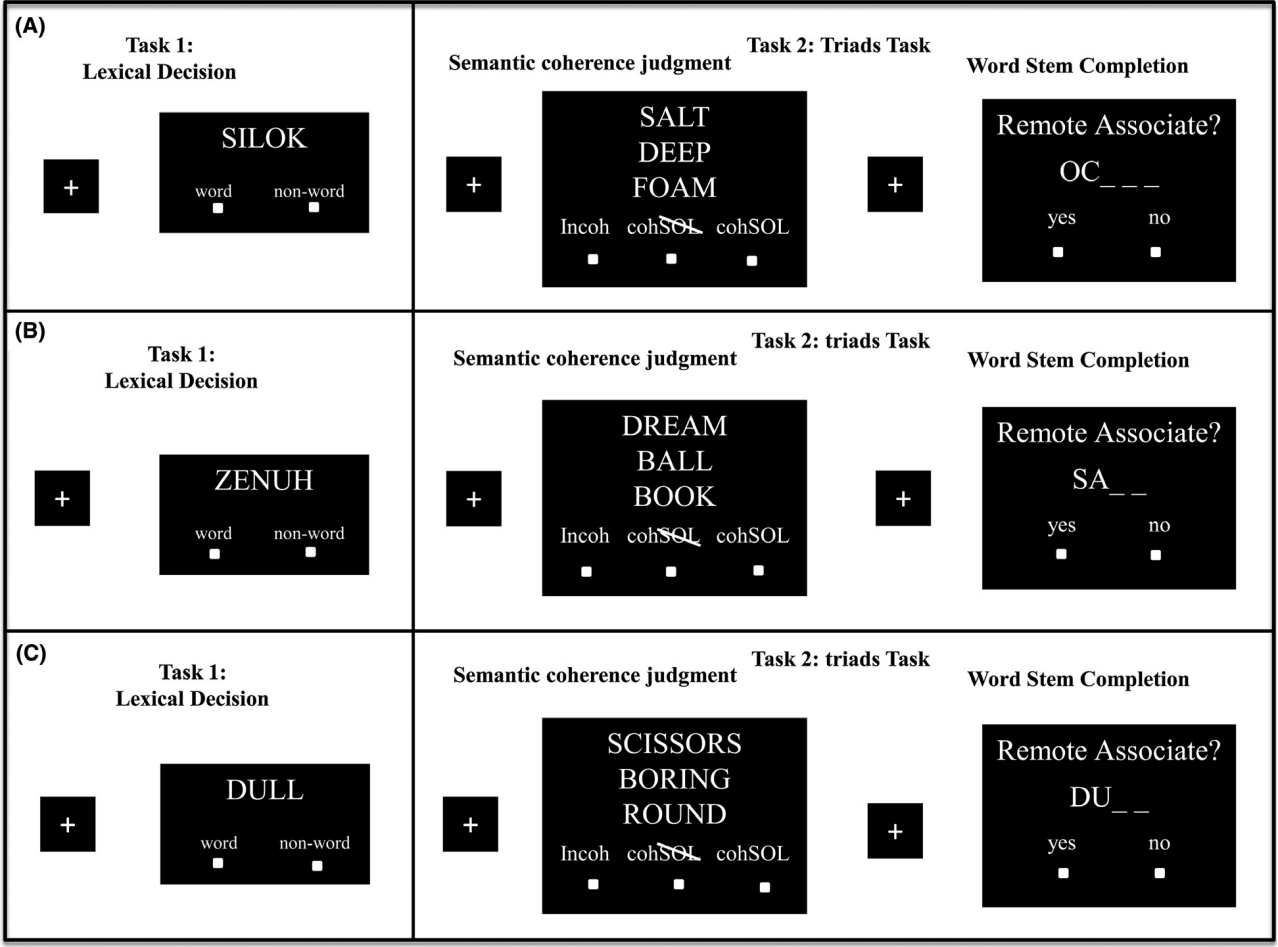
Experimental design fMRI study. Participants worked on alternating blocks of lexical decisions and the triads task, whereby the triads task consisted of semantic coherence judgments and word‐stem completions. In the coherence judgment task, participants had three response options: incoh = the triad is perceived as incoherent (response option 1: “The triad is incoherent”); coh SOL = the triad is perceived as coherent, but a possible CA cannot be named immediately (response option 2: „The triad is coherent and therefore has a fourth word in common, but a CA cannot be retrieved at this time”); and cohSOL = the triad is perceived as coherent, and a possible CA can be named immediately (response option 3: “The triad is coherent and a CA can be retrieved immediately”). To test whether participants could name the correct CA when they had judged the triad as coherent and at the same time indicated that they knew the CA, they were presented with all coherent and primed triads again right after the scanning procedure and had to write them down in a paper‐and‐pencil questionnaire. (A) Example of a coherent triad preceded in the lexical decision block by either a non‐word or a semantically unrelated word, and followed in the word‐stem completion by the first two letters of the actual solution. (B) Example of an incoherent triad preceded in the lexical decision blocks by either a non‐word or an unrelated word, and followed in the word‐stem completion by the first two letters of a semantically unrelated word. (C) Example of a primed triad preceded in the lexical decision blocks by the prime (i.e., consisting of the synonym of one word of the three triads constituents), and followed in the word‐stem completion by the first two letters of this primed synonym (target).

The experiment consisted of two different experimental blocks: (1) lexical decision blocks (i.e., incorporation of a conceptual priming procedure),^2^ and (2) semantic coherence judgments blocks (i.e., usage of the triads task to assess intuitive performance). Please consult Figure 2 for an overview of the experimental design.

In the lexical decision blocks (eight in total, each consisting of 20 trials), participants were presented every 2 sec with a word or a non‐word and had to decide which of the two it was. This block basically functioned as conceptual priming. There were three types of lexical decision tasks: First, primes were synonyms of one of the triad words of the incoherent triads that were presented in the subsequent coherence judgment block. It is important to note that solution concepts (i.e., CAs) of coherent triads were never used as primes. Second, non‐primes consisting of words that were semantically unrelated to all other words were used as fillers. These non‐primes were self‐made. Third, non‐words were used to create a meaningful task for the participants. These non‐words were taken from Ilg et al. (2007). All in all, 20 stimuli for which participants had to make a lexical decision were presented in each block. These 20 stimuli consisted of primes (synonyms of words of incoherent triads), non‐primes or fillers (words that were semantically unrelated to all of the subsequent triad words) and non‐words (letter strings that were meaningless in and of themselves but were pronounceable in principle). The combination of primes, non‐primes/fillers, and non‐words, as well as the order in which they appeared, were randomized for each participant.

In the semantic coherence judgment blocks (eight in total, each consisting of 15 trials), each trial consisted of two parts: the coherence judgment and a subsequent word‐stem completion. First, the word triad was presented (all three words presented simultaneously, one beneath the other), and participants had to judge its semantic coherence within 4 sec.

They had three response options:

□ “The triad is incoherent” (defined in the instructions as not having any word in common) (response option 1),
□ “The triad is coherent and therefore has a fourth word in common, but a CA cannot be retrieved at this time” (response option 2), and
□ “The triad is coherent and a CA can be retrieved immediately” (response option 3).

To test for a (dis‐)continuity model of intuition, we manipulated the original response format of the triads task (traditionally usage of two response options: coherent versus incoherent), in which participants are asked to assess the semantic coherence of word triads. That is, by adding a third response option, we were able to map the participants’ answers onto the two‐stage model of intuition (cf. Bowers et al. 1990). Participants were instructed to rely on their feeling as to whether or not the three clue words of a triad belonged together semantically and, if so, whether they could name the triad's CA. It was made clear to the participants that they did not need to immediately know a CA in order to indicate a triad as semantically coherent (they could choose response option 2 in such cases). Regardless of participants’ specific responses (1, 2, or 3), they were subsequently presented with a word‐stem‐completion task for 3 sec. Participants were asked (in a “yes/no” format) to indicate whether or not the letters shown were the first two letters of the CA they had had in mind during the previous semantic coherence judgment. In the case of coherent triads, the word‐stem completion began with the first two letters of the preordained solution concept; in the case of incoherent triads, the word‐stem completion began with random letters; and in the case of primed triads, the word‐stem completion began with the first two letters of the synonym with which that triad had been previously primed in the lexical decision task. Then the next trial began.

**Figure 3:**
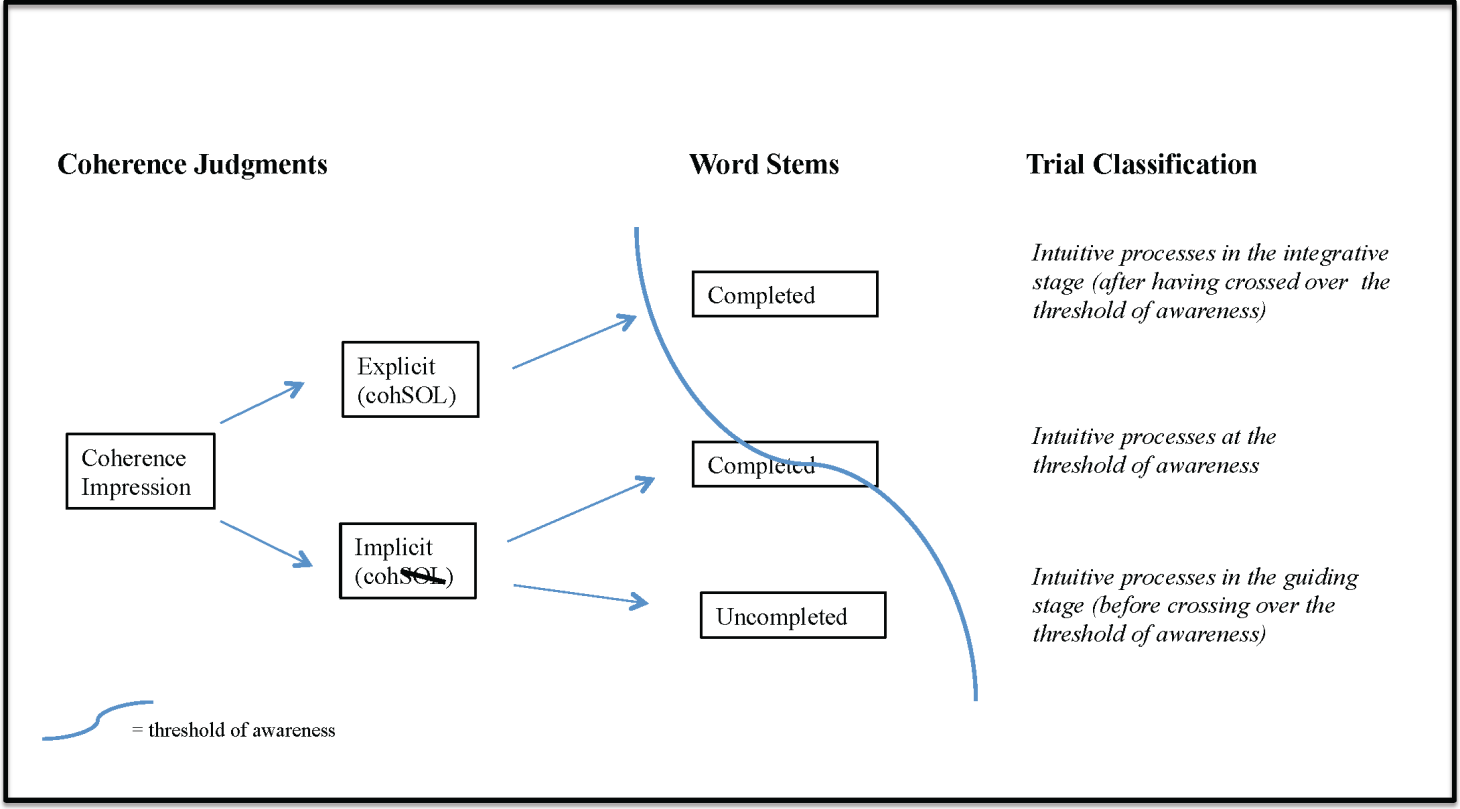
Gradual mapping onto the two‐stage model of intuition for coherent triads. According to Bowers et al. (1990), intuitive processing can occur within two different stages. The *guiding stage* is rather implicit since the source of the coherence impression cannot be explicitly verbalized. The *integrative stage*, however, is rather explicit since the coherence impression can now be consciously accessible and consequently, the source of the impression can be explained. The transition from one stage to the other is assumed to be fostered by the accumulation of activated concepts in semantic memory automatically driven by environmental clues. In our trial classification, we stick to this theoretical account and mapped the participants’ responses onto the two stages of the model. By adding a third response option (i.e., splitting coherence judgments into implicit and explicit ones in addition to incoherence judgments) as well as by means of the word‐stem completions subsequently following the coherence judgments, we were able to classify each trial with respect to whether the participant has already crossed the threshold of awareness. Explicit (cohSOL) = response option 3: “The triad is coherent and a CA can be retrieved immediately”. Implicit (cohSOL): response option 2: “The triad is coherent and therefore has a fourth word in common, but a CA cannot be retrieved at this time”.

By using a three‐part response scheme and then the subsequent word‐stem completions, we, for the first time, directly implemented – at the time of the coherence judgment – a graded mapping onto a two‐stage model following Bowers et al. (1990) conceptualization of intuition. Since it was, for our purposes, particularly important to determine which of the two stages a participant was in and how close to the threshold of awareness that moment was, we discerned three kinds of intuitive processes for coherent triads (i.e., our trial classification for the behavioral and imaging analyses):

- *Intuitive processes in the guiding stage (before overcoming the threshold of awareness)* denoted by responses where participants in the coherence judgment chose option 2 AND indicated in the word‐stem completion that they did not know the CA.
- *Intuitive processes at the threshold of awareness* denoted by responses where participants in the coherence judgment chose option 2 AND indicated in the word‐stem completion that they knew the CA; and
- *Intuitive processes in the integrative stage (after having crossed the threshold of awareness)* denoted by responses where participants in the coherence judgment chose option 3 AND indicated in the word‐stem completion that they knew the CA (see Fig. 3).

Imaging data analyses focused on the blocks of the semantic coherence judgment task and ignored the lexical decision blocks and word‐stem completions. The former served for priming only, the latter for validating the priming and determining the intuitive processing type and thus the trial classification as explained above.

With our stimulus material and task design, all trials could be objectively classified into three types of triads: *coherent* (45 triads)*, incoherent* (30), or *primed* (45).^3^ Objectively coherent trials were those where a coherent triad was presented in the semantic coherence judgment task. Objectively incoherent trials were those where an incoherent triad was presented in the semantic coherence judgment task. And primed trials were those where incoherent triads were presented in the semantic coherence judgment task AND were preceded by a synonym of one of the three clue words in the lexical decision task given prior to the coherence task (see Fig. 2). Hence the whole priming procedure had two phases (as it is typical for priming experiments): First, in the lexical decision task, participants were primed with a concept. Then, in the coherence judgment, they encountered a similar stimulus. It is highly likely that a facilitating effect from the first to the second encounter of the stimulus would be observed as a consequence. Significantly, it was ensured that none of the words presented in the lexical decision task were semantically related to or a synonym of any of the preordained CAs of the coherent triads presented in the subsequent semantic coherence judgment block. By taking only synonyms from the clue words of incoherent triads as primes (to constitute the priming condition), we ensured that CAs were generated by the participant not because of the triads internally priming the solution (cf. behavioral pre‐study on the ASA effect) – as it is the case with coherent triads – but because of the conceptual priming procedure (externally applied by our experimental design) only. In this way, it was possible to keep intuition‐based decisions separate from priming‐based decisions, which served our main goal in research question 2 of addressing a neural disentanglement of intuition and implicit memory.

Since the study's validity highly depends on the stimulus material, we set great value on stimulus generation. The generation of the stimulus set used, its validation as well as a full overview of all used triads is given in the supplement provided online (Appendix S1: Pre‐studies 1–3; Appendix S2: List of coherent and incoherent word triads; Appendix S3: List of primed word triads).

We asked participants to name a word in the post‐scan questionnaire (in which they were presented with all of the coherent and primed triads that had appeared during the scanning session) in order to ensure their not having missed any potential CAs. In this way, we could enable an exact classification of solved and unsolved coherent triads (Bolte et al. 2003). This post‐scan questionnaire was used for a consistency check between answers given in the semantic coherence judgments, in the word‐stem completions, and in the naming of solution concepts after scanning. In particular, we compared answers given in the word stems and in the post‐scan questionnaire to find out whether participants were consistent in indicating to know or not to know a possible CA in both measures. We expected that in case a participant answered with a “yes” in the word‐stem completion during scanning was also able to solve the respective coherent triad in the post‐scan questionnaire, that is to write down the correct CA (cf. Results section).

### Experimental procedure and MRI data acquisition

When participants arrived at the scanner, they were informed both orally and in writing about details of the MR scanning procedure and the experimental task. They were then placed inside the MR scanner, wearing protective earplugs to mitigate the strong noise that the scanner produces. To map the correct location of a participant's head, an initial localizer spanning a few seconds was run at the beginning of the scanning procedure. The experimental task started after localization and lasted approximately 20 min. The final part of the scanning procedure consisted of acquiring anatomical images, which took 10 min.

After having left the MR scanner, the participant immediately filled out the post‐scan questionnaire, in which all the triads from the coherent and primed conditions were shown again, and for which the participant had to come up with an appropriate solution concept or any word that came to mind at that moment. Retrieval of these solutions was performed immediately after the scanning in order to gather as many CAs as the participant might have come up with during the experiment.^4^

Functional magnetic resonance imaging took place at the Max Planck Institute for Biological Cybernetics in Tübingen. Image recording was performed with a 3 Tesla MRI scanner (SIEMENS MAGNETOM Trio Tim syngo MR B15). A standard head coil was used. Since we expected activity within the OFC, and, as has been repeatedly noted in the literature, this area is prone to suffer from signal losses (due to any strong susceptibility gradients near air/tissue interfaces), we used a spin‐echo (SE) sequence (Deichmann et al. 2002; Balteau et al. 2010). Although SE sequences are known to be less sensitive to susceptibility artifacts, a major disadvantage of this technique is its lower statistical power (Norris 2012). Seventeen slices (3 mm thickness, distance factor (gap) 10%, field of view [FOV] 210 mm, data matrix of 3.3 × 3.0 voxels, and base resolution of 64) were acquired. Repetition time amounted to 2000 msec and time to echo was 88 msec. To allow for measurements to be taken at numerous time points along the blood‐oxygenation‐level‐dependent signal curve, we jittered both the onset of the coherence judgment as well as the time between the coherence judgment and the word‐stem completion task. That is, the onset of each triad presentation (coherence judgment) was relative to the beginning of the first of seven scans and varied randomly in four time steps (10, 500, 1000, and 1500 msec). The time between coherence judgment and word‐stem completion task could randomly be either 3500, 4500, or 5500 msec. Thus, the length of each trial was 14 sec (coherence judgment = 4 sec, word‐stem completion task = 3 sec, jitter between consecutive trials = max. 1.5 sec, and jitter between judgment and stem completion within each trial = max. 5.5 sec.). The purpose of this procedure was to enhance the temporal resolution of the imaging acquisition (Miezin et al. 2000; Birn et al. 2002). Participants were unaware of this modulation. Since we measured only 17 slices in a SE sequence that, on the one hand, was particularly suited to detect a potential OFC activation, it was, on the other hand, not possible to find activation in posterior and parietal areas.

### Image analysis

The MRI data were processed using the software package FSL (FMRIB's software library, www.fmrib.ox.ac.uk/fsl), version 4.1.9. First, some pre‐analyses were carried out as follows: Functional data were motion‐corrected using a rigid‐body registration to the central volume (Jenkinson et al. 2002). With the BET brain extraction tool, images were then separated from substances that did belong to the actual brain of the participant (i.e., layers of skin, etc.) (Smith 2002). In order to compensate for the temporal shift between the different slices measured within one TR of 2000 msec, a sinc interpolation correction was used (slice time correction). To eliminate signals with low frequencies, a high‐pass filter with a cut‐off frequency of 1/100 Hz was applied. A spatial smoothing was carried out using a Gaussian filter of 5 mm FWHM (Full‐Width Half‐Maximum). The registration of each individual's EPI‐images with her structural high‐resolution images, as well as the normalization to the standardized spatial orientation (MNI space, Montreal Neurological Institute), was done via the fsl tool FLIRT (Jenkinson and Smith 2001; Jenkinson et al. 2002). This overlaying process enabled averaging across distinct group means since the anatomical structures of each individual brain were adjusted with the coordinates of the standard MNI brain.

The statistical evaluation was based on a general linear model (GLM) using FILM (FMRIB's improved linear model) for the individual analyses. Contrast images were calculated separately for each individual by estimating the raw‐score differences between specified conditions. These single‐subject contrasts were entered into a second‐level group analysis. The auto‐correlations of measurements were taken into account by using a pre‐whitening procedure. An event‐related design was implemented, that is, the hemodynamic response function (HRF) was modelled in terms of the experimental conditions for each stimulus (event = onset of triad presentation). Furthermore, the HRF was defined by a double gamma function and its first derivative. The explanatory variables were as well modelled with its temporal derivatives. To take the differences in response latencies into account, we modelled the amplitude of each explanatory variable by RT, that is each trial was modelled individually by RT. Thus we could rule out that different neuronal activation patterns between the conditions resulted from differences in the response latencies indicating different degrees of difficulty in the triads (i.e., easiness/difficulty to explicitly name a CA). We used a corrected cluster significance threshold of *P *=* *0.05 and determined clusters by *Z* > 2.3 to threshold the *Z* statistic images (Worsley 2001).

## Results

All behavioral analyses were carried out with the statistics software SPSS (SPSS Statistic 20.0; IBM, Chicago, IL). Missed trials were not included in the analyses. The imaging analysis was done with FSL (FMRIB, Oxford, U.K.).

### Lexical decision task

To validate that the participants had paid attention to our conceptual priming procedure and thus had had the chance to process the primes by working on them, we tested whether participants were correct in discriminating between words and non‐words in the lexical decision task. Our results showed that participants correctly classified 96.38% (SD = 2.46) of the words as words and 98.44% (SD = 1.79) of the non‐words as non‐words. Thus participants performed the lexical decision task correctly, that is, they cognitively processed the primes, which was the main goal that we pursued with the lexical decision task. The mean RTs of the lexical decision task were 837.45 msec (SD = 87.74) for words recognized as words, and 876.57 msec (SD = 70.05) for non‐words recognized as non‐words, a significant difference (*t *=* *3.56, df = 18, *P *<* *0.002, two‐tailed). It is well‐known that words are recognized more quickly than non‐words due to frequency effects, where frequently occurring combinations of letter strings prompt faster recognition (Gardner et al. 1987; Wagenmakers et al. 2004); we were able to replicate that result and can conclude that participants worked on the primes and processed them correctly.

### Behavioral results hypothesis 1: response pattern and intuition index

Table 2 shows the response behavior of participants in the semantic coherence judgment task, separated for objectively coherent, objectively primed, and objectively incoherent triads: Participants were for the most part correct when judging objectively coherent triads as (subjectively) coherent (74.68% of the objectively coherent triads) and objectively incoherent triads as (subjectively) incoherent (51.19% of the objectively incoherent triads). Of these subjective coherence judgments, 43.44% were implicit, that is, participants indicated that they did not know the CA at the time of the coherence judgment, and 31.24% were explicit, that is, participants indicated that they knew the CA immediately. Data from the post‐scan questionnaire revealed that the majority of explicit answers (response option 3), were correct (70.73%) – in other words, participants had provided the correct CA or a synonym of that word. To check whether participants were consistent in their answers (i.e., that they both answered with a “yes” in the word‐stem completion and then were able to solve the respective coherent triad in the post‐scan questionnaire), we performed a consistency check between word‐stem completion and post‐scan questionnaire data. Analysis revealed that participants were by and large consistent: When indicating that they knew the CA in the word‐stem completion, they could also most of the time solve the triad in the post‐scan questionnaire (62.88%). Thus coherent triads were regarded as solved if participants answered with a “yes” in the word‐stem completion task, in that way indicating that they knew the CA. On average, 22.95% of the 45 objectively coherent triads were considered solved in that sense.

**Table 2:** Response behavior for coherent, primed and incoherent triads in percent with standard deviations in parentheses

	Coherent	Primed	Incoherent
Response option 1	25.30 (± 11.02)	52.52 (± 21.07)	51.19 (± 15.08)
Response option 2	43.44 (± 18.90)	38.93 (± 18.59)	37.18 (± 16.86)
Response option 3	31.24 (± 19.73)	8.54 (± 10.14)	11.61 (± 10.07)

Response option 1 = “The triad is incoherent”.

Response option 2 = “The triad is coherent and therefore has a fourth word in common, but a CA cannot be retrieved at this time”.

Response option 3 = “The triad is coherent and a CA can be retrieved immediately”.

In order to detect the participants’ ability to decide intuitively, the *intuition index* was computed following Bolte and colleagues (2003). The *intuition index* is defined as the hit rate (i.e., the proportion of unsolved coherent triads that are nevertheless correctly classified as being coherent) minus the false alarm rate (i.e., the proportion of incoherent triads that are incorrectly classified as coherent) when the CA is unknown.^5^ In our paradigm, *this intuition index can be computed* in two ways: Variant “a” takes both of the answers indicating an impression of coherence into account (response options 2, and 3), as long as the following word‐stem task cannot be solved, whereas variant “b” considers only response option 2. Consequently, it is possible to determine a person's ability to intuitively decide in two ways: a broader measure that would include both answers indicating an impression of coherence, or a narrower one, which is only possible by applying the three‐part response scheme. The especialness is, that both variants represent two different levels of unknowingness here. Variant “a” represents the case where participants either think that they knew the solution concept but were still wrong or when they know that they did not know the solution. Variant “b” represents only answers where participants knew that they did not know the solution.

Table 3 shows hitrates, false alarm rates, and the resulting intuition indices for both variants. Both indices indicate that participants were able to intuitively decide in the given task: Participants decided above chance level whether the three clue words belonged together, even if they could not come up with the CA. Thus with both intuition indices, we could replicate previous findings on that task (Bowers et al. 1990; Bolte et al. 2003; Bolte and Goschke 2005; Ilg et al. 2007; Topolinski and Strack 2008; Remmers et al. 2014).

**Table 3:** Hitrate, false alarms and intuition index in percent for both variants with standard deviations in parentheses

	Hitrate	False alarms	Intuition index
Variant “a”	63.58 (± 16.95)	48.8 (± 15.08)	14.77 (± 13.92)
Variant “b”	44.72 (± 16.95)	37.18 (± 16.86)	7.53 (± 11.45)

For variant “a”, unsolved coherent triads were determined based on two properties: participants rated the triad as coherent (response option 2 or 3) BUT did not subsequently complete the word‐stem (i.e., they answered with a “no” to indicate that they did not know the CA). The false alarm rate consisted of incoherent triads that participants rated as coherent (response option 2 or 3) BUT for which they did not subsequently complete the word‐stems. Hit rate and false alarm rate differed from the chance level of 33.3 (hitrate: *t*(18) = 7.86, *P *<* *0.001; false alarm rate: *t*(18) = 4.56, *P *<* *0.001). The intuition index departed significantly from 0 (*t*(18) = 4.62, *P *<* *0.001).

For variant “b”, unsolved coherent triads were determined based on two properties: participants rated the triad as coherent without knowing a CA (response option 2) BUT did not subsequently complete the word‐stem (i.e., they answered with a “no” to indicate that they did not know the CA). The false alarm rate here consisted of incoherent triads that participants rated as coherent (response option 3) BUT for which they did not subsequently complete the word‐stems. The hit rate differed from the chance level of 33.3, the false alarm rate did not differ from chance level (hit rate: *t*(18) = 3.01, *P *<* *0.001; false alarm rate: *t*(18) = 1.08, *P *=* *0.293). The intuition index departed significantly from 0 (*t*(18) = 2.86, *P *=* *0.01).

### Behavioral results hypothesis 1: reaction time data of the coherence judgment and the word‐stem completion

Since potential (confounding) RT effects were important to know for the set of regressors in the fMRI analyses, we tested for those. It was an exploratory analysis insofar as previous studies on the triads task either did not find any differences or did not report any. For the RTs of the coherence judgment, we first ran an ANOVA with *type of triad* (coherent versus incoherent versus primed) and *judgment* (response options 1, 2 and 3) as within‐subjects factors, which yielded significant overall main effects of *type of triad* (*F*(2, 36) = 4.63, *P *=* *0.016.) and *judgment* (*F*(2, 36) = 13.57, *P *=* *0.000), as well as a significant *interaction* between the two variables (*F*(4, 72) = 6.07, *P *=* *0.000). The main effect of *type of triad* showed that coherent triads were answered faster than incoherent ones. The main effect of *judgment* revealed that answers, where participants immediately indicated to know the CA were the fastest trials. The interaction effect is especially interesting with respect to the objectively coherent triads: The RT in trials where participants chose response option 3 (i.e., the triad is coherent and a CA can be immediately retrieved) (*M* = 2813.23, SD = 278.48) was faster than the RT in trials where they chose response option 2 (i.e., the triad is coherent, but a CA cannot be retrieved immediately) (*M* = 2905.31, SD = 211.66), which itself was faster than the RT in trials where participants chose response option 1 (i.e., the triad is incoherent) (*M* = 3100.58, SD = 301.40), a movement that may be a first behavioral hint, empirically revealed, of the gradual nature of intuitive semantic coherence judgments (Table 4A).

**Table 4:** Reaction times of the coherence judgments in milliseconds with standard deviations in parentheses: (A) Reaction times dependent on the triad's condition (coherent, primed, incoherent), and (B) Reaction times for the three discerned kinds of intuitive processes mapped onto the two‐stage model (coherent triads only)

	Coherent	Primed	Incoherent
(A)			
Response option 3	2813.23 (± 278.48)	1733.82 (± 137.40)	2307.32 (± 258.34)
Response option 2	2905.31 (± 211.66)	3052.60 (± 278.79)	3046.86 (± 217.56)
Response option 1	3100.58 (301.40)	3060.13 (± 189.63)	3055.83 (± 334.14)

	Coherent		
(B)			
After threshold crossing	2782.18 (± 349.28)		
At threshold crossing	2862.86 (± 217.29)		
Before threshold crossing	2914.18 (± 271.13)		
Incoherence Judgment	3100.58 (± 301.40)		

Response option 1 = “The triad is incoherent”.

Response option 2 = “The triad is coherent and therefore has a fourth word in common, but a CA cannot be retrieved at this time”.

Response option 3 = “The triad is coherent and a CA can be retrieved immediately”.

After threshold crossing = Intuitive processes in the integrative stage (denoted by responses where participants in the coherence judgment chose option 3 AND indicated in the word‐stem completion that they knew the CA).

At Threshold crossing = Intuitive processes at the threshold of awareness (denoted by responses where participants in the coherence judgment chose option 2 AND indicated in the word‐stem completion that they knew the CA).

Before threshold crossing = Intuitive processes in the guiding stage (denoted by responses where participants in the coherence judgment chose response option 2 AND indicated in the word‐stem completion that they did not know the CA).

To determine whether this gradual decrease in RTs (i.e., *increased response speed*) is observable for the three discerned kinds of intuitive processes as well (i.e., a gradual mapping onto a two‐stage model), we also looked at this trial classification (see description in the methods section). We statistically compared the RTs of coherent triads for the *intuitive processes in the guiding stage (before overcoming the threshold of awareness)*, the *intuitive processes at the threshold of awareness*, the *intuitive processes in the integrative stage (after having crossed the threshold of awareness)*, and the *incoherence judgment* with each other. Again, a gradual decrease can be seen in the RTs. The highest values (i.e., slowest RTs) occurred for incoherence judgments (*M* = 3100.58, SD = 301.40), and the RTs steadily descended from there. Intuitive processes in the guiding stage of intuition (when having not yet crossed over the threshold of awareness) showed slightly faster response speeds (*M* = 2914.18 (SD = 271.13)), followed by intuitive processes at the threshold of awareness (2862.86 (SD = 217.29)), and finally, intuitive processing in the integrative stage of intuition (when having crossed the threshold of awareness) showed the fastest response speeds (*M* = 2782.18 msec, SD = 349.28). A repeated measurements ANOVA with the factor *intuitive processes* revealed that the values differed significantly from each other (*F*(3, 54) = 5.27, *P *=* *0.003) (Table 4B).

### Imaging results hypothesis 1: intuition – a gradual process

In order to test whether intuitive coherence judgments in the guiding stage qualitatively or quantitatively differ from intuitive coherence judgments in the integrative stage, we compared them with regard to their neural correlates (19 participants). Testing hypothesis 1, we used a design with five regressors: (1) Intuitive processes in the guiding stage (response option 2 AND participants indicated that they did not know the CA in the word‐stem completion); (2) Intuitive processes at the threshold of awareness (response option 2 AND participants indicated that they knew the CA in the word‐stem completion); (3) Intuitive processes in the integrative stage (response option 3 AND participants indicated that they knew the CA in the word‐stem completion); (4) Incoherence judgments: Trials, where participants judged incoherent triads as incoherent (response option 1); and (5) Primed trials: all incoherent triads that had been preceded by a synonym of one of their triad constituents in the lexical decision blocks, irrespective of what the participants answered in the coherence judgment and in the word‐stem completion (since this was not a regressor of interest for research question 1).

**Figure 4:**
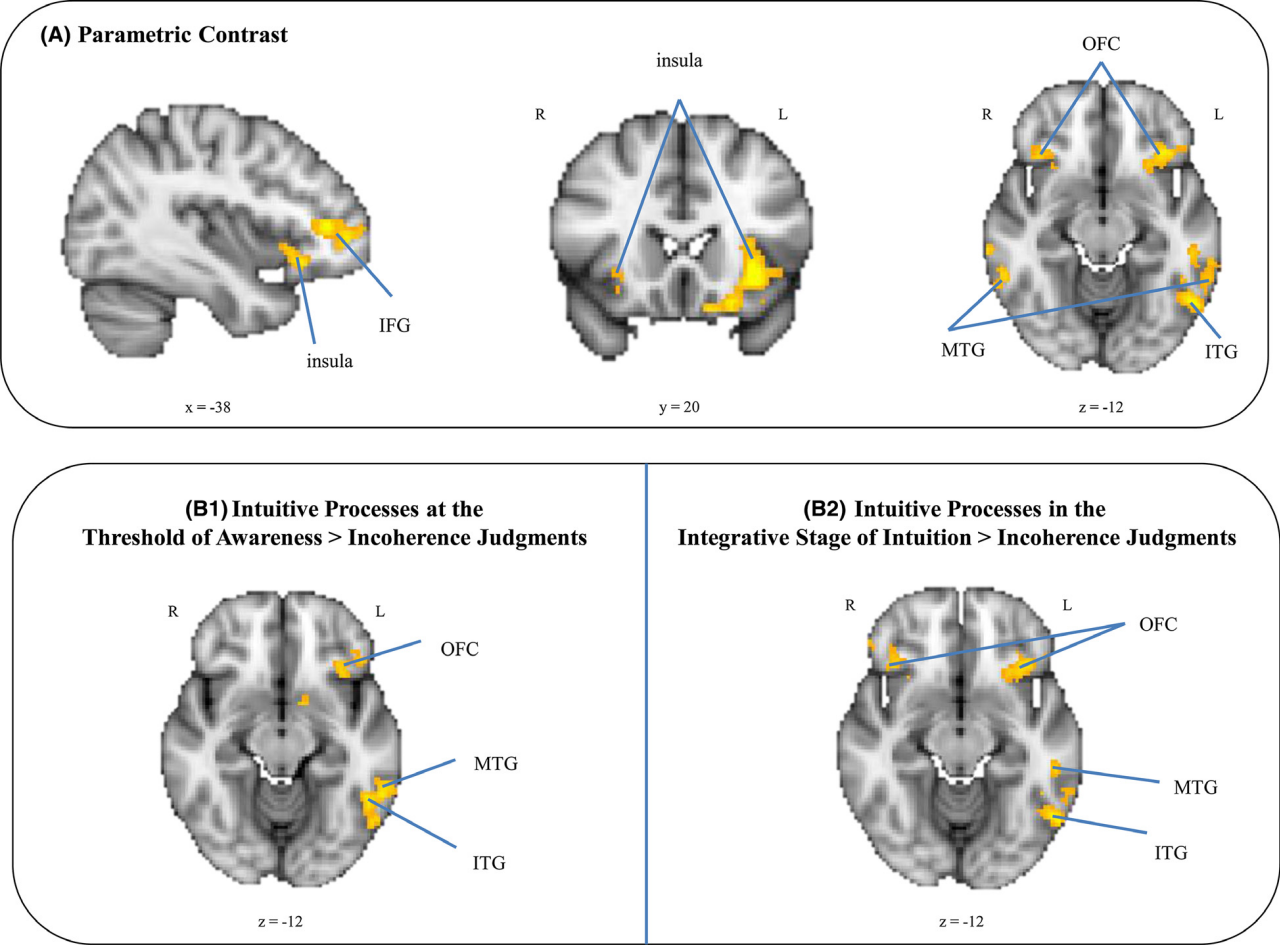
Imaging results research question 1. Group‐averaged significant activation patterns on coronal, sagittal, and axial slices of an individual brain normalized and aligned to the Talairach stereotactic space are shown. (A) Parametric contrast. (B_1_) Contrast: Intuitive Processes at the Threshold of Awareness > Incoherence Judgments. (B_2_) Contrast: Intuitive Processes in the Integrative Stage of Intuition > Incoherence Judgments. IFG = inferior frontal gyrus, ITG = inferior temporal gyrus, MTG = middle temporal gyrus OFC = orbito‐frontal gyrus.

We first analyzed a parametric contrast in order to explore a possible increase in instances of perceived incoherence from instances of perceived coherence where a CA immediately comes to mind. In parametric contrasts, a continuous increase that is linearly modelled is assumed. This means that all trials within one condition (i.e., in each of our five regressors) are considered equal and at the same time are considered either lower or higher than all trials within the other conditions. Results revealed a bilateral activation within the posterior OFC, within the insula, within the left IFG extending into the frontal pole, and within the left posterior part of the MTG. The temporo‐occipital part of the left inferior temporal gyrus (ITG) and the anterior median prefrontal cortex (mPFC) were activated as well; the latter comprised the pregenual anterior cingulate cortex (ACC, BA 32) and extended into the anterior part of the medial prefrontal cortex (mPFC) (BA 10) (Table 5 and Fig. 4A).

**Table 5:** Laterality, anatomical specification, MNI space coordinates and Z values of peak voxels of activated clusters for the parametric contrast (research question 1) are shown

Area	*x*	*y*	*z*	*Z*
Inferior frontal gyrus	−38	40	8	3.73
Orbitofrontal cortex	−32	20	−10	3.56
	−22	22	−18	3.56
Anterior insula	−32	22	−6	4.1
Middle temporal gyrus	−62	−38	−8	4.1
Inferior temporal gyrus	−52	−60	−10	3.69
	−54	−62	−14	3.52
Pregenual ACC	−2	48	8	3.65
	6	40	10	3.58
	4	48	4	3.46

This gradual increase in activation within the described network was also visible when we examined the individual contrasts alone, that is, when we contrasted the different kinds of intuitive processes against incoherence judgments. Specifically, the contrast between intuitive processes at the threshold of awareness and incoherence judgments revealed a left‐sided network of activation within the OFC, the insula, the IFG (extending into the frontal pole) and the MTG and ITG (Fig. 4b_1_). Importantly, this left‐sided network expanded to the right side, revealing bilateral activation in the OFC, the insular cortex, and the IFG when contrasting intuitive processes in the integrative stage of intuition against incoherence judgments (Fig. 4b_2_). For the contrast between intuitive processes in the guiding stage of intuition (before having crossed the threshold of awareness) and incoherence judgments we found the same left‐sided network showing activity in the OFC, the insular cortex, IFG, MTG, and ITG but only when we had lowered the statistical threshold to *Z* > 2.0

### Behavioral results hypothesis 2: priming effects

Primed triads are defined as incoherent triads one word of which was preceded by its synonym in the lexical decision task executed beforehand. As reported in the section *Response Pattern and Intuition Index* (cf. Table 2)*,* participants in the fMRI study did not indicate the majority of the primed triads as coherent (i.e., did not choose response option 2 or 3), which means that not every single participant showed the intended priming effect in the scanner. This result was rather astonishing, since we had run a behavioral pilot study prior to the fMRI study to ensure that the intended priming effect would be possible in principle. In this behavioral priming pilot, seven participants (three female, mean age 23 years, SD 2.51, range 19–27), none of whom participated in the fMRI study and in the reported behavioral pre‐study investigating the ASA, behaviorally performed the experiment (lexical decision blocks alternating with semantic coherence judgment blocks) and did show the priming effect. For the coherence judgment, these participants indicated 84.03% of the primed triads as coherent (59.83 with response option 2 and 24.2% with response option 3). To go into more detail, every single participant in this pilot study showed the priming effect, that is, they all indicated with response option 2 or 3 above‐chance primed triads as coherent (participant 1: 92.1%; participant 2: 92.1%; participant 3: 92.1%; participant 4: 82.05%; participant 5: 81.26%; participant 6: 75.68%; and participant 7: 72.97%). We interpreted this result as evidence of a successful priming procedure, stopped piloting after the seven participants showed a priming effect and moved the actual experiment to the scanner.

To check whether at least some of the participants showed the intended priming effect in the scanner, we analyzed the response pattern in the coherence judgment of primed triads individually for every single subject of the fMRI study. By doing so, we could determine seven participants out of the 19 who showed a successful priming effect in that they indicated 70.53% (SD = 10.64) of the primed triads as coherent (participant 1: 85.19%; participant 2: 81.48%; participant 3: 73.53%; participant 4: 70% participant 5: 64.71%; participant 6: 64.71%; participant 7: 54.17%). Furthermore, for all participants, trials where they chose response option 3 were the fastest RTs in all three conditions (coherent, incoherent and primed) (Table 4B). Accordingly, in the priming condition as well, the RTs for response option 3 were the fastest ones and were even faster than in the other two conditions. This result indicates that primed triads were processed faster than coherent and incoherent trials, which is taken as additional evidence that those seven participants processed the primes correctly and perceived the triad as more coherent than non‐primed (objectively) incoherent triads, presumably due to the previous encounter in the lexical decision task. Thus we took the data of only these seven participants to answer research question 2 on the neural level. We took these results as a first preliminary hint, interpreting them with due care.

### Imaging results hypothesis 2: intuition – a primed process

To test whether intuition‐based and priming‐based decisions are qualitatively or quantitatively different on a neuronal level, we compared the neural correlates of intuition‐based decision processes with those of priming‐based decision processes (seven participants, each of whom successfully showed the priming effect in the scanner). Testing hypothesis 2, we analyzed the data from those seven participants who successfully showed a priming effect in the scanner and used a design with five regressors: (1) Intuitive processes in the guiding stage; (2) Intuitive processes at the threshold of awareness; (3) Intuitive processes in the integrative stage; (4) Incoherence judgments; and (5) Successfully primed triads: primed triads that participants indicated as coherent (response option 2 or 3). The main difference between the regeressors used to answer research question 1 and the regressors used to answer research question 2 was regressor (5). To answer research question 1, we used all primed triads in regressor (5), since this was not a regressor of interest here. To answer research question 2, we used only trials, where the priming procedure was successful in that participants misattributed semantic meaning to primed triads (i.e., they indicated primed triads as coherent). To enable an analysis of the data of these seven participants, we lowered the threshold to *Z* > 2.0 for all contrasts reported below.

**Figure 5:**
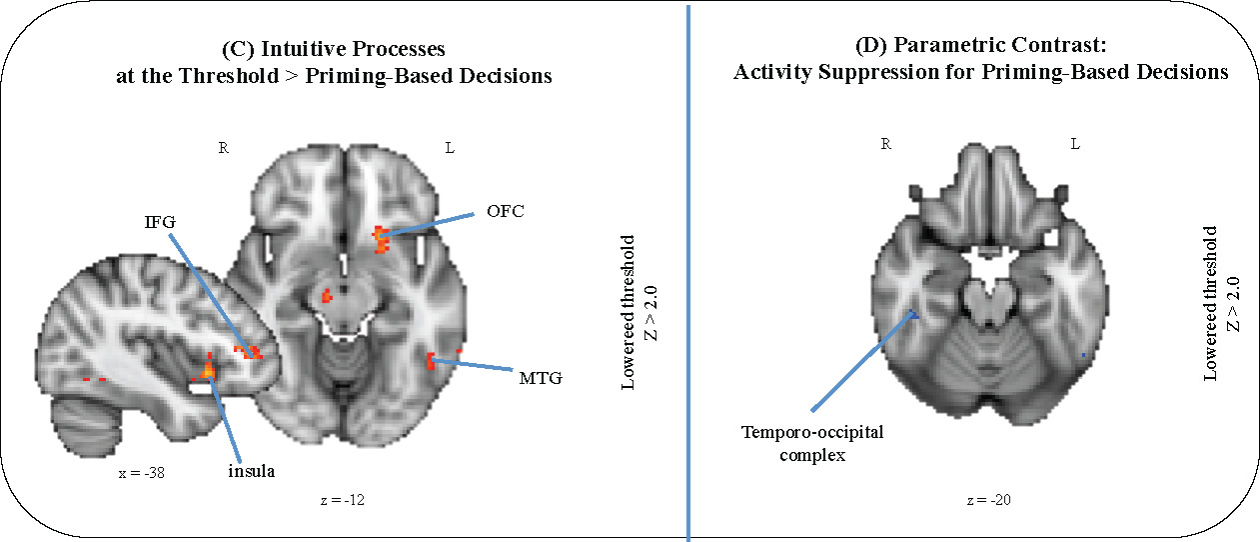
Imaging results research question 2. Group‐averaged significant activation patterns on coronal, sagittal, and axial slices of an individual brain normalized and aligned to the Talairach stereotactic space are shown. (C) Contrast: Intuitive Processes at the Threshold > Priming‐Based Decisions. (D) Parametric contrast: Activity suppression for priming‐based decisions. IFG = inferior frontal gyrus, MTG = middle temporal gyrus OFC = orbito‐frontal gyrus.

To test whether intuition‐based and priming‐based decisions elicit different activation patterns, we compared the three different kinds of intuitive processes with the successfully primed trials and vice versa. Primed trials did not show any specific activation in this comparison ((5) vs. (1); (5) vs. (2); (5) vs. (3)). That is, for primed trials, we did not observe any specific activation pattern when contrasted with intuitive trials. As opposed to this, when we contrasted intuition‐based decisions against priming‐based decisions we found a brain network specifically activated in this contrast. To compare intuition‐based and priming‐based decisions directly, we contrasted intuitive processes at the threshold of awareness with priming‐based decisions ((2) vs. (5)), since this contrasts suited best for our aim to compare the two concepts. In case of priming‐based decisions, the priming was externally induced via our research design. Contrary to that, processes at the threshold of awareness reflect some kind of an internal priming processing, namely when the three clue words of a coherent triad converge on a common concept. Results revealed activation within the left posterior OFC (*x* = −20, *y* = 16, *z* = −14), within the left ITG (*x* = −48, *y* = −56, *z* = −14) and within the right ventral tegmental area (*x* = 10, *y* = −18, *z* = −10) for intuitive processes at the threshold of awareness ((2) vs. (5)) (Fig. 5C).

To replicate previous findings on the neural correlates of conceptual priming processes, we specifically investigated an activity suppression for priming‐based decisions ((5)). When looking at the parametric activation, results revealed activity suppression in the right temporo‐occipital cortex (*x* = 48, *y* = −54, *z* = −16) – that is, in the occipital fusiform gyrus and a tiny (i.e., small in region) activation in the temporal occipital fusiform cortex – for priming‐based decisions (Fig. 5D).

## Discussion

In the present fMRI study we focused on an automatic‐spread‐of‐activation (ASA) account, within which intuitive processing has been conceptualized as occurring within two different stages (Bowers et al. 1990). In the first guiding stage, participants have a strong impression of coherence triggered by environmental clues that initiate the automatic activation of semantic concepts in memory. Albeit being strong enough to act upon, the source of this impression is not yet consciously available. A gradual accumulation of activated concepts, however, may lead to (semantic) activation crossing over a threshold of awareness. As a consequence, participants are then able to verbalize their coherence impression, which has been defined as occurring within the second integrative stage of intuition (cf. Bowers et al. 1990).

Following this framework, we pursued our first research question, namely: Is the gradual nature of intuitive decision making put forward by Bowers et al. (1990) in their two‐stage model reflected on a neural level of cognitive processing? Or does the abrupt awareness of a mental product or end state (cf. solution concept) rather reflect a genuine discontinuity in the underlying perceptual‐cognitive processing of information?

The conceptualization of intuition as a non‐conscious process exerting influence on behaviour by drawing on implicitly acquired knowledge that signals higher processing areas in the conscious brain resembles the definition of implicit memory (cf. Volz and Zander 2014). Thus, in our second research question we asked whether intuition‐based and priming‐based decisions share the same neural substrates or whether they rather differ qualitatively. To answer both research questions, we conducted an fMRI study and let participants assess the semantic coherence of word triads, thereby using intuition‐based and priming‐based decisions elicited by the same material in the same participants for the first time.

Behaviorally, we could replicate the finding that participants were able to reliably discriminate between coherent and incoherent triads above chance level (Bowers et al. 1990; Bolte and Goschke 2005; Ilg et al. 2007; Topolinski and Strack 2008; Remmers et al. 2014). Moreover, we could demonstrate that applying a three‐part response scheme (instead of the two response options “coherent” and “incoherent” that have been traditionally used) captures the proposed gradual nature of intuitive decision making and enables to detect people's tendency to decide intuitively. Thereby, we went one step further assessing already at the time of the coherence judgement, that is, at the earliest time point, whether a potential CA is popping up in the participants’ mind. This was essential for the investigation of the proposed gradual nature of intuitive semantic coherence judgments.

Regarding research question 1, we found a left‐sided brain network activated for intuitive answers comprising the posterior part of the OFC, the insula, the IFG (extending into the frontal pole), the ITG, and the posterior division of the MTG. Most interestingly, this activated network increased gradually (i.e., quantitatively) from instances of perceived incoherence to instances of perceived coherence. More precisely, activation expanded into the right OFC and right IFG for intuitive answers, where participants correctly judged the coherence and at the same time were aware of a common denominator of this coherence impression (i.e., when they were in the integrative stage of intuition explicitly naming the correct CA).

Regarding research question 2, we can only draw preliminary conclusions, since out of our 19 participants only seven showed the intended priming effect in the scanner. This stands in contrast to a behavioral priming pilot where all participants showed a priming effect. Possible causes for this discrepancy between the behavioral performance and the performance in the scanner are suggested to be global ones, such as a distraction by the scanner's noise and/or a higher arousal when being in the scanner that might have prevented a priming effect. The laying position in the scanner might also have had an effect on the performance. These causes, however, are valid for all fMRI experiments and are thus not specific for our investigation. It has to be noted, however, that the possibility of a positive priming effect's occurrence recently came under fire. Shanks et al. (2013) for example, based on their findings on an attempt to replicate the results of well‐known priming experiments, argue that positive priming effects are elusive phenomena, if it all possible to induce. If priming effects occur, they are very short‐lived and hard to grasp, the authors propose. Thus, it may not be too surprising that not every participant showed a priming effect in the scanner, where the external conditions may have aggravated the probability to obtain an effect as compared with an experimental setting outside the scanner (for an overview of this debate see also Yong 2012). Nevertheless, when we lowered the threshold to calculate statistics, we observed activity within the left posterior OFC and the left ITG for intuition‐based decisions contrasted to priming‐based decisions. We additionally were able to replicate previous conceptual priming results in that we found activity suppression in the right temporo‐occipital complex as correlate of priming‐based decisions. Future studies will be necessary to (1) discover the reasons why participants in some cases perform the same behavioral task (i.e. in this case the triads task) differently outside and inside the scanner, and (2) to further disentangle potential overlapping and distinct neural activation patterns of intuitive decision‐making mechanisms and implicit memory processes.

With this contribution we hope to advance the still ongoing theoretical debate as well as neurobiological theorizing on intuitive processing by providing intriguing results on the gradual nature of intuitive coherence processing as well as preliminary evidence for a clear neuronal distinction between intuition and priming. The findings are discussed and embedded in existing literature in the following.

### Intuitive processes in the guiding stage differ quantitatively from processes in the integrative stage

For research question 1, data speak in favour of the continuity model of the underlying perceptual‐cognitive processes for the type of task used (even if the solution phenomenological seems to surface discontinuously as a sudden insight). The gradual (quantitative) increase of activation within the reported brain network (comprising the OFC and IFG) is taken to reflect the continuous build‐up of coherent information that will then cross a threshold of awareness or noticing. Accordingly, this result is at the same time taken to mitigate the assumption of a discontinuity model of intuition, in which a more or less spontaneous restructuring of the problem representation may yield its solution – conditions suggested to be typical for insight problems (cf. Ohlsson 1992; Knoblich and Öllinger 2006; Kounios and Beeman 2014). Yet, in tasks such as the one used here, no rule learning or reorganization processes are necessary to find a solution. Instead, one has to probe one's memory network and wait for a (positive) response that will be occurring when semantic activation elicited by cues in the environment converges on a common concept (i.e., when environmental cues activate highly and remotely associated concepts). This is in line with conclusions drawn by previous findings on this task that all rest on the conceptualization of intuitive judgments within an automatic‐spread‐of‐activation account (Bowers et al. 1990; Bolte and Goschke 2005; Ilg et al. 2007). According to this account, knowledge is represented in terms of nodes and associative pathways between the nodes. When part of the network is activated, for instance by reading a word triad, activation spreads along the associative pathways to (semantically) related areas in memory; and thus eventually also leads to the activation of the CA. Bowers et al. (1990) elucidate: “[A] common associate is more likely to be multiply activated when it has a common meaning with respect to each of the three clue‐words” (p. 80). In other words, “the greater the associative‐spreading along the associative connections is (Collins and Loftus 1975; Anderson 1983), the greater is the activation “echo” from associated units back to the target item's unit (Nelson et al. 1998)” (Hofmann et al. 2011, p. 2).

This process can now be further specified by our RT data: Results of our behavioral pre‐study on the ASA and the RT pattern of our imaging study both suggest that the longer one has to wait for a (positive) response from the activation echo, the more likely it will be that the triad is considered incoherent (cf. incoherence judgments take the longest). In contrast, relatively quick responses about the association strength will rather lead to intuitive responses, even if the final output is not the solution concept. As the ASA pre‐study could demonstrate, the longer one has to wait for making a response, the more likely it is that the coherence will not be detected as such. The advantage of the automatically spreading activation in semantic memory is its rapid onset. The results of the ASA pre‐study may indicate a slowing down of the ASA after some time coupled with a loss of advantage for being non‐consciously susceptible to the detection of coherence. The RT pattern in the fMRI study revealed that trials where participants immediately know the CA are the fastest trials, whereas responses, for which participants correctly judge coherence but without knowing the CA are slower. In other words, it reflects the intuitive processing of environmental meaning (i.e., being sensitized to detect meaning in the environment without yet being able to justify this hunch) towards an explicit representation. The finding that trials, where participants are able to verbalize a CA are the fastest trials nicely show that point in the intuitive processing chain when after the tacit and non‐conscious intuitive pre‐processing the recognition of a CA surfaces into consciousness – an, according to Bowers et al. (1990), for the person seemingly sudden event that nevertheless has gradually build up over time. Remember, that RT differences were controlled for in the fMRI analysis therewith variance due to RT differences was factored out.

Our imaging results support the conceptualization of a continuity model of intuitive judgments within an automatic‐spread‐of‐activation account. For intuition‐based decisions at the threshold of awareness, we observed a left‐sided brain network of activity within the OFC, the insular cortex, the IFG (extending into the frontal pole), ITG, and the posterior part of the MTG, a network that conforms to previous findings in the language processing literature. In particular, these areas have been discussed in the context of semantic processing of graphematic (visual) stimuli and verbal working memory processes, especially when the search for a solution or a related concept is ensued (Bookheimer 2002; Gernsbacher and Kaschak 2003). Thus the observed network mainly reflects the semantic processing initiated by the three clue words in our task. Particularly, it here reflects the semantic readout processing (i.e., figuring out the meaning) of the three clue words and the lexical search for a solution (i.e., the CA). More activation within this brain network is taken to reflect greater activation echo, for instance when this echo comprises activation of the solution concept.

We would like to highlight particularly the involvement of the IFG in language processing, especially in the decoding of the word meaning in phrases. In her review, Bookheimer (2002) explains that the IFG “appears to represent a unique brain region involved not in decoding meaning of individual words but in processing semantic relationships between words or phrases, or in retrieving semantic information”. Semantic processing of visual stimuli and the search for a solution concept of the triads is thus reflected in our data by the activated left‐sided brain network, which is in accordance with previous results (Bookheimer 2002; Gernsbacher and Kaschak 2003; Ilg et al. 2007).

One region that is especially interesting when investigating intuition‐based decision processes is the OFC. The OFC has been suggested as a candidate region for intuitive processing that, due to the high number of (inter)connections to and with other brain areas, serves as first integrator of (incomplete) incoming stimuli (Bar et al. 2006; Volz and von Cramon 2006; Volz et al. 2008; Luu et al. 2010; Horr et al. 2014). In a recent MEG study, Horr et al. (2014), in their preliminary neural model of intuitive processing, specify that the OFC receives its input from early sensory areas. It then functions, across domains, as the global integrator of the incomplete or vague stimulus input delivering an initial interpretation that can be perceived as a preliminary hunch about the nature of the stimuli. After this coarse representation, where the gist of the information is extracted, the information is transferred to further domain‐specific areas where a more detailed interpretation of the content takes place. Our data further support this model; thus we are able to extend it with respect to the following aspects: As shown, previous studies observed OFC activation particularly as correlate of an intuitive “integration process of unconsciously represented information” (Volz and von Cramon 2006, p. 2) when the incoming input is incomplete, vague, or ambivalent. This could be shown so far for visual (Volz and von Cramon 2006) and auditory stimuli (Volz et al. 2008). On the basis of our data we are now able to augment the understanding of the neural underpinnings of intuitive processing by two further aspects: Firstly, we could show that the OFC's function as global and gist‐extracting integrator is also valid in the semantic domain, in cases when participants are asked to intuitively judge the coherence of ambivalent word triads (ambivalent since the three clue words may trigger different solutions). Secondly, our data support the two‐stage model of intuition put forward by Bowers et al. (1990) on a neural level, showing activation patterns that expand from the guiding stage of intuition to the integrative stage of intuition insofar as the left‐sided activation networks becomes bilateral (i.e., extending into the right cortex).

Another region we found activated for intuition‐based decisions is the anterior insular cortex. The anterior insula is involved in a plethora of cognitive functions in the domain of subjective feelings and self‐awareness (e.g., self‐recognition, awareness of body movement, making a smile, subjective cooling, and attention to heat pain (for an overview see for instance Craig 2009)). Moreover, the insular cortex has been recently discussed in the context of interoception, a concept that refers to the feelings that we perceive from our bodies (Craig 2002, 2009; Critchley et al. 2004). According to Craig (2002), it is the “sense of the physiological condition of the body” (p. 655). In the task used here, activation within the insula may reflect the subjective *feeling* of coherence accompanying the coherence judgment. To explicate, the three clue words of the triad, when spreading semantic activation had converged on a solution concept, elicits an intuitive impression of coherence that is perceptible as a strong drive or tendency towards a specific option. This *sensing/feeling* component of the coherence impression may be reflected by activation in the anterior insular cortex.

### Intuitive processing and implicit memory

For research question 2, preliminarily, we found suppressed activity in the right temporo‐occipital complex. Importantly, these area does not overlap with the areas that are activated in intuition‐based decisions, namely we did not find any OFC activation, nor activity within the anterior insular cortex, IFG, the MTG nor the IFG. Instead, the deactivation pattern found for priming‐based decisions reflects previous results on priming processes. There has been consensus that priming is based on a facilitation of perceptual processes that manifests itself in an improvement of performance and in an acceleration of response speed. Neurally, this facilitation appears in suppressed activation patterns as correlate for the second encounter with the primed stimulus (e.g., Squire et al. 1992; Schacter et al. 1998).

Our results fit into this picture thereby showing that intuition‐based and priming‐based decisions did not rest on the same brain networks. It may be, however, thinkable that implicit memory mechanisms are a prerequisite for an intuition to occur. Coherence detection in the task used here would not be possible without implicit experiences with the material. As Bowers et al. (1990) point out: “[T]hese relatively automatic processes are embedded in and emerge out of the personal history and experience of a given individual” (p.93). Along these lines, Kihlstrom et al. (1996) elaborate: “The presentation of a problem, or a retrieval cue, activates and integrates relevant preexisting knowledge structures, and in the course of solving the problem, or remembering the event, the cognitive system builds on these structures, accruing new information and gradually approaching the solution” (p. 18). In other words, deciding intuitively whether a word triad converges on a fourth concept is (non‐consciously) driven by pre‐existing knowledge of semantic relatedness of all three words, thereby internally priming the solution.

The present data on the difference between intuitive processing and priming, however, have to be treated with due care and are only taken as a preliminary hint in the direction of intuitive decision‐making mechanisms and implicit memory processes differing in their neural correlates, which in turn may suggest that the two concepts differ substantially on a theoretical level. Nevertheless, future studies are needed to further investigate the (inter)relationship between intuition‐based and priming‐based decisions.

## Conflict of Interest

None declared.

## Supporting information


**Appendix S1.** Generation of Stimulus Material: Pre‐Studies 1–3.

**Appendix S2.** List of coherent and incoherent word triads.

**Appendix S3.** List of primed word triads.

## Notes

1 Please note that Bowers et al. (1990) description of the two‐stage model may not be entirely free from ambiguity and may also allow a somewhat different interpretation than ours. In this contribution the correct detection of environmental meaning yet without being able to justify this hunch is taken to reflect early intuitive processing in the guiding stage, and the explicitly verbalized justification of this hunch in form of a solution to a given problem is taken to reflect processes in the integrative stage of intuition. Yet, we are also aware of a different connotation: Some authors may regard the first kind of answers as being already indicative for the integrative stage stating that the recognition of coherence becomes explicit and not necessarily its justification in form of a solution. Thus, in other studies of intuition, explicitly solved trials were typically excluded from the analyses (e.g., Topolinski and Strack 2009a).

2 Lexical decision tasks have frequently been used to investigate priming processes (e.g., Meyer and Schvanefeldt 1971; Scarborough et al. 1977; Wagenmakers et al. 2004). This can be done in two different ways: (1) For usage as primes, lexical decisions have to be performed in the first phase of a priming experiment as a first encounter with the to‐be‐primed item (2) For usage as targets to directly test the priming effect, lexical decisions have to be performed in the second phase of a priming experiment as the second encounter with the already‐primed stimulus. Here, a facilitating effect in response to this second encounter has been typically observed. In our fMRI study, we used lexical decisions as primes as the first manner described.

3 This uneven split was used since incoherent triads only served as control stimuli.

4 Due to the danger of strong movement artifacts, we did not gather CAs by the participants orally communicating it to us during scanning. To nevertheless be able to collect CAs, we asked for these in the post‐scan questionnaire.

5 Please note that the intuition index has been frequently used to study the sensitivity of intuitive decision making processes (Bolte and Goschke 2005; Ilg et al. 2007; Topolinski and Strack 2008; Remmers et al. 2014). It has its origin in signal detection analysis and has the following functions: (1) assessing intuitive performance and (2) correcting for potential response biases (by means of the false alarm rate). It can therefore discriminate between participants who assign coherence to each triad and participants who correctly differentiate between coherent and incoherent triads (i.e., who perform intuitive semantic coherence judgments).
