# Supplementary material for: Intuitive decision making as a gradual process: investigating semantic intuition‐based and priming‐based decisions with fMRI
Source: Brain Behav. 2015 Dec 22;6(1):e00420. doi: 10.1002/brb3.420 (PMC4834943; doi:10.1002/brb3.420)
Supplement: Supplementary file 1 — Appendix S1. Generation of Stimulus Material: Pre‐Studies 1–3. [file BRB3-6-e00420-s001.docx]

Appendix S1. Generation of Stimulus Material: Pre-Studies 1-3.

We began with a stimulus set of 200 word triads, 60 of them kindly provided by Bolte, Goschke, and Kuhl (2003). We developed the rest of the word triads ourselves by using material from Bowden and Jung-Beeman (2003) as a template; these were translated and with slight changes constructed to allow for German language regularities. All triads were predefined objectively as either semantically coherent or semantically incoherent on the basis of the work of Bolte, Goschke, and Kuhl (2003) and Bowden and Beeman (2003). A semantically coherent triad is characterized by the fact that its constituents converge on a fourth concept that is commonly connected to the meaning of all three words of the respective triad. An example of a coherent triad is: SALT, DEEP, FOAM. The common solution concept (i.e., the common associate CA) for this triad is SEA or a synonym for SEA. In contrast, semantically incoherent triads do not converge on such a fourth word that the original three constituents of the triad have in common. An example of an incoherent triad is: FINGER, SPEAK, WORM.

To empirically validate and thereby select our final stimulus material from all of the triads, both those provided by Bolte and colleagues (2003) and those that we devised, we run three pre-studies. In *Pre-study 1*, 24 participants (18 female, mean age 25.95 years, SD 1.72, range 20-27) were presented with all triads (in randomized order) and had to indicate whether or not they perceived each triad to be coherent. Of the triads that were predefined as coherent, 80.09% were rated as coherent; of the triads that were predefined as incoherent 91.60% were rated as incoherent. Based on these results, we adjusted our stimulus material, deleting all objectively coherent (incoherent) triads that were rated as incoherent (coherent) by more than 65% of the participants. For the remaining triads, a t-test revealed that participants rated predefined coherent triads as coherent and predefined incoherent triads as incoherent (*t*(23) = -17.08, *p* = .000). We call these triads objectively coherent and objectively incoherent, respectively. Our final set of word triads used for the fMRI study, then, consisted of 45 objectively coherent and 75 objectively incoherent triads.

To ensure that these 120 selected objectively coherent and incoherent triads differed only in the semantic relatedness of the three clue words and the CA and not in the pairwise semantic relatedness of the three clue words, we ran two further pre-studies (2 and 3) that exactly followed the procedure developed by Bolte and Goschke (2005, cf. p. 1249).

In *Pre-study 2*, we had 15 participants (7 female, mean age 25.73, SD 4.65, range 22-39) who had not participated in Pre-study 1 rating the semantic relatedness of the three clue words and the CA for both objectively coherent and incoherent triads on a 5-point scale ranging from 1 (not at all semantically connected) to 5 (strongly semantically connected). For objectively coherent triads, we presented preordained CA words based on Bolte, Goschke and Kuhl (2003) and Bowden and Beeman (2003). For objectively incoherent triads we presented entirely unrelated words, or words that only matched the semantic category of one constituent of an incoherent triad. The mean of these ratings was 3.84 (SD = .36) for the coherent triads and 1.66 (SD = .44) for the incoherent triads, values that were reliably different (*t*(14) = 22.10, *p* < .001). Thus this result revealed, as intended, that the mean semantic relatedness of the clue words and their CAs was reliably higher for the coherent triads than the mean for the incoherent triads. Based on these results, we did not exclude any triads from the material set. Instead, we were successful in showing that the CAs of our stimulus material were indeed perceived as strongly connected to the corresponding objectively coherent triads, and vice versa – that objectively incoherent triads were not perceived as connected to any word.

In *Pre-study 3*, 15 participants (8 female, mean age 25.73 years, SD 4.65, range 22-39) who had not participated in either Pre-study 1 or 2 rated the semantic relatedness between pairs of clue words, that is, three pairings for each triad (e.g., PURE-BLUE, BLUE-FALL, PURE-FALL), again on a 5-point scale ranging from 1 (not at all semantically connected) to 5 (strongly semantically connected). The mean of these pairing ratings was 2.32 (SD = .61) for the coherent triads and 2.11 (SD = .62) for the incoherent triads, values that did not differ significantly [*t*(60) = 1.30, *p* = .19], which was as intended. According to Bolte and Goschke (2005), "[One] must make sure that the relatedness among the three clue words is not higher for coherent than for incoherent triads" (p. 1249) so as not to attribute the performance of the participants to objective triad features differing between conditions but rather to the association of the clue words with their common remote associate. The authors explain: "Otherwise, above-chance judgments could be based on the (explicit) perception of associations among the clue words rather than the (unconscious) activation of the common solution concept" (Bolte & Goschke, 2003, p. 1249). Again, we did not need to exclude more triads; through the pre-test, we ensured that the three single-word pairs of an objectively coherent triad were not perceived as more semantically connected than the three single-word pairs of an objectively incoherent triad.

We further ensured that the words used in the objectively incoherent and coherent triads of our stimulus material did not differ in number of letters (*t*(217) = -.69, *p* = .488), or syllables (t(217) = -.27, p = .788.), concreteness (t(217) = -1.20, p = .23), frequency with which they might be used in daily language (t(217) = 1.66, p = .09), or number of phonemes (t(217) = -.27, p = .78). Having thus validated our triads according to the above criteria we kept our final stimulus set consisting of 45 objectively coherent and 75 objectively incoherent triads (cf. Supplement 2 and 3).
