# Supplementary material for: Intuitive decision making as a gradual process: investigating semantic intuition‐based and priming‐based decisions with fMRI
Source: Brain Behav. 2015 Dec 22;6(1):e00420. doi: 10.1002/brb3.420 (PMC4834943; doi:10.1002/brb3.420)
Supplement: Supplementary file 2 — Appendix S2. List of coherent and incoherent word triads. [file BRB3-6-e00420-s002.doc]

Appendix S2. List of coherent and incoherent word triads. List of used stimuli showing type of triad (coherent = coh, incoherent = inc), each word of the triad plus its English translation (italic, in brackets) and the preordained CA of coherent triads (i.e., the CA). Please note that all triads were shown in German. Thus, the English translation may not adequately reflect the semantic associations spreading out from each word of the triad in every single case.

Type of Triad Word 1 Triad Word 2 Triad Word 3 Common[[1]](#footnote-2)

Triad Associate (CA)

Coh HEIZUNG EIS WINTER KALT

(*heater*) (*ice*) (*winter*) (*cold*)

Coh ZERBROCHEN KLAR AUGE GLAS

*(broken) (clear) (eye) (glass*

Coh COMPUTER RENNEN RAKETE SCHNELL

*(computer) (race) (rocket) (quick)*

Coh PAPPE QUADRAT OFFEN SCHACHTEL *(paperboard) (square) (open) (cardboard box)*

Coh GROß BLATT SCHATTEN BAUM

*(big) (leaf) (shadow) (tree)*

Coh SALZ TIEF GISCHT MEER

*(salt) (deep) (foam) (sea)*

Coh BLUETE FALTEN EURO SCHEIN

*(phony) (folding) (euro) (banknote)*

Coh STOFF ELEND KARTOFFEL SACK

*(stuff) (misery) (potatoe) (sack)*

Coh DECKEL LESEN PAPIER BUCH

*(cover) (reading) (paper) (book)*

Coh LOCHER TESA LAMPE SCHREIBTISCH

(hole punch) (sellotape) (lamp) (desk)

Coh FLUG ENGPASS HOCHWERFEN OBEN

(flight) (shortage) (tossing) (up)

Coh GELD STUHL KELLNER RESTAURANT

*(money) (chair) (waiter) (restaurant)*

Coh TROPISCH WILDHUETER ERHALTEN REGENWALD

*(tropical) (gamekeeper) (preserve) (rainforest)*

Coh BATTERIE STRAHL ZELTEN TASCHENLAMPE

*(battery) (beam) (camping) (torch)*

Coh FASS GARTEN BAUCH BIER

*(barrel) (garden) (belly) (beer)*

Coh RIEMEN TASCHE ZEIT UHR

*(strap) (bag) (time) (watch)*

Coh TROEDELN GEHEN SIRUP LANGSAM

*(loitering) (walking) (syrup) (slow)*

Coh MANIEREN RUND TENNIS TISCH

*(manners) (round) (tennis) (table)*

Coh GLAS STROM HELL GLUEHBIRNE

*(glass) (electricity) (bright) (lightbulb)*

Coh TOENUNG HAAR OEL FRISEUR

*(hair tint) (hair) (oil) (hairdresser)*

Coh ROHR HOLZ HEIß OFEN

*(pipe) (wooden) (hot) (oven)*

Coh MOEBEL BAUM FEUER HOLZ

*(furniture) (tree) (fire) (wood)*

Coh SALAT TOPF GABEL ESSEN

*(salad) (pot) (fork) (eating)*

Coh WURM REGALBRETT STUETZE BUCH

*(worm) (shelf) (bookend) (book)*

Coh EDEL RAUSCH WASCHEN GOLD

*(noble) (rush) (washing) (gold)*

Coh KISSEN LEDER WOHNZIMMER SOFA

*(pillow) (leather) (living room) (sofa)*

Coh FALLE POLARLICHT KLAUE EISBAER

(trap) (polar lights) (claw) (ice bear)

Coh VOGEL SEITEN BUTTER GELB

*(bird) (pages) (butter) (yellow)*

Coh TROCKEN KOPF HUND KNOCHEN

*(dry) (head) (dog) (bones)*

Coh KANINCHEN WOLKE SAHNE WEIß

*(rabbit) (cloud) (cream) (white)*

Coh TRAUM ANBRUCH LICHT TAG

*(dream) (dawn) (light) (day)*

Coh TIEF STEIN WASSER BRUNNEN

*(deep) (stone) (water) (well)*

Coh HERZ FEUER AMPEL ROT

*(heart) (fire) (traffic light) (red)*

Coh ZITRONE SAND MOND GELB

*(lemon) (sand) (moon) (yellow)*

Coh SPIELEN KREDIT GRUß KARTE

*(playing) (credit) (greeting) (card)*

Coh WASSER TROPEN DAMPF FEUCHT

*(water) (tropes) (steam) (humid)*

Coh ZEIT HAAR STRECKEN LANG

*(time) (hair) (stretch) (long)*

Coh ESSEN VORWAERTS PAUSE WANDERN

*(eating) (forward) (break) (hiking)*

Coh SCHNEIDE AMPUTATION LANGWEILIG STUMPF

*(grind) (amputation) (boring) (blunt)*

Coh NATUR FREUND PFADFINDER ZELTEN

*(nature) (friend) (scout) (camping)*

Coh SCHUHE SHIRT SCHNELL LAUFEN

*(shoes) (teeshirt) (fast) (jogging)*

Coh HIMMEL WASSER TINTE BLAU

*(sky) (water) (ink) (blue)*

Coh SCHMERZ JAEGER KOHL KOPF

*(pain) (hunter) (cabbage) (head)*

Coh FADEN PINIE SCHMERZ NADEL

*(twine) (pine) (ache) (needle)*

Coh LAERM QUALM HEIß FEUER

*(noise) (thick smoke) (hot) (fire)*

Inc MAEDCHEN SOZIAL HORN

*(girl) (social) (cow horn)*

Inc KAMMER MASKE NATUERLICH

(cabin) (mask) (natural)

Inc STAB HERSTELLER PUNKT

*(rod) (producer) (dot)*

Inc KNALL- FLIEGE KAEMPFER

BONBON

*(firecracker) (fly) (fighter)*

Inc SCHWER FARBEN STOSS

*(heavy) (colours) (shove)*

Inc GUERTEL HANDEL NASE

*(belt) (trade) (nose)*

Inc NAGEL FADEN SPIELEN

*(nail) (thread) (playing)*

Inc MUEHLE ZAHN STAUB

*(mill) (tooth) (dust)*

Inc RECHT KATZE KOHLE

*(law) (cat) (coal)*

Inc GOLD CRACKER RAD

*(gold) (cracker) (bike)*

Inc VOGEL ROHR STRAßE

*(bird) (pipe) (street)*

Inc MESSER FUß SCHACHTEL

*(knife) (foot) (box)*

Inc VOGEL HERING DAMPF

*(bird) (herring) (steam)*

Inc VORSTELLUNG RUDERN LEBEN

*(imagination) (rowing) (life)*

Inc BEWEGUNG BLAU TASCHE

*(movement) (blue) (bag)*

Inc SICHERHEIT ISSEN PUNKT

*(safety) (pillow) (dot)*

Inc SCHLAF BOHNE MUELL

*(sleep) (bean) (rubbish)*

Inc BONBONS GIEBEL STADT

*(candies) (pediment) (town)*

Inc MAGIE BELLEN KREDIT

*(magic) (bark) (credit)*

Inc HAUS LOEWE BUTTER

*(house) (lion) (butter)*

Inc KLAR ROLLEN MACHT

*(clear) (rolls) (might)*

Inc STADT WURZEL AUTO

*(city) (root) (car)*

Inc SALBEI FARBE SCHREIBEN

*(sage) (colour) (writing)*

Inc KADETT KAPSEL SCHIFF

*(cadet) (capsule) (ship)*

Inc VERLIERER KEHLE PLATZ

*(loser) (gorge) (place)*

Inc KEKSE LEER ZUEGEL

*(biscuits) (empty) (reins)*

Inc SCHWAN ARMEE MASKE

*(swan) (army) (mask)*

Inc TROCKEN LADEN TENNIS

*(dry) (shop) (tennis)*

Inc FRANZOESISCH AUTO SCHUH

*(french) (car) (shoe)*

Inc SEIDE STEIN ZIEGE

*(silk) (stone) (goat)*

1. Please note that the preordained CAs indicate *one* plausible answer that would have been counted as “correct solution” for the respective triad; other synonyms were also counted as correct when semantically making sense in an individual case. Complete CAs were not presented to the participants at any time. [↑](#footnote-ref-2)
