# Supplementary material for: Intuitive decision making as a gradual process: investigating semantic intuition‐based and priming‐based decisions with fMRI
Source: Brain Behav. 2015 Dec 22;6(1):e00420. doi: 10.1002/brb3.420 (PMC4834943; doi:10.1002/brb3.420)
Supplement: Supplementary file 3 — Appendix S3. List of primed word triads. [file BRB3-6-e00420-s003.doc]

Appendix S3. List of primed word triads. List of primed triads showing each word of the triad plus its English translation (italic, in brackets) and the prime that had been shown to the participant in the lexical decision blocks prior to the coherence judgments. Primes were synonyms of one triad word of incoherent triads. These words are marked in bold font. Please note that all primes and triads were shown in German. Thus the English translation may not adequately reflect the conceptual priming.

Type of Triad Word 1 Triad Word 2 Triad Word 3 Prime

Triad

Imp **HAUS** DECKE BALL HEIM  *(house) (blanket) (ball) (home)*

Imp **BOTTICH** HAUCH RAUM ZUBER

*(vat) (breeze) (chamber) (tub)*

Imp **SCHULD** SCHRANK KUMPEL FEHLER

*(guilt) (wardrobe) (mate) (fault)*

Imp ESSEN FRONT- **DROGE** RAUSCHGIFT

SCHEIBE

*(food) (front window) (drug) (narcotic)*

Imp **MEISTER** WERFEN FINGER KOENNER

*(master) (throwing) (finger) (adept)*

Imp BUCHT **ERKER** GURKE BALKON

*(bay) (oriel) (cucumber) (balcony)*

Imp STAPEL MARKT **ZIMMER** RAUM

*(pile) (market) (chamber) (room)*

Imp **GLAESERN** FROEHLICH EILE DURCHSICHTIG

*(vitreous) (cheerful) (hurry) (transparent)*

Imp WELPE WAHR **BRIEF** SCHREIBEN

*(puppy) (true) (letter) (writing)*

Imp **ROLLEN** BOHNE SCHIFF KUGELN

*(rolling) (bean) (ship) (bowling)*

Imp SCHNELL LOEFFEL **BILDSCHRIM** MONITOR

*(fast) (spoon) (screen) (display)*

Imp ALTER MEILE **SAND** STRAND

(age) (mile) (sand) (beach)

Imp ERBSE **SCHALE** BRUST SCHUESSEL

*(pea) (bowl) (chest) (tureen)*

Imp **FETZEN** MILCH UNKRAUT LUMPEN

*(rag) (milk) (weed) (tatter)*

Imp TRINKEN **KLINGEL** RUECKEN SCHELLE

*(drinking) (bell) (back) (clamp)*

Imp **VORWAERTS** SPUELUNG RASIERER GERADEAUS

*(ahead) (flushing) (shaver) (straight on)*

Imp **ZAHENE** ARREST START GEBISS

*(teeth) (detention) (start) (ivories)*

Imp GRAS **KOENIG** FLEISCH HERRSCHER

*(grass) (king) (meat) (ruler)*

Imp BOHNE UNTERBRECHUNG **KUCHEN** TORTE

*(bean) (recess) (cake) (pie)*

Imp LOEFFEL TUCH KARTE KELLE

*(spoon) (scarf) (card) (ladle)*

Imp NASS RECHT **GESCHAEFT** LADEN

*(wet) (law) (business) (shop)*

Imp TOMATE **ZEMENT** ZAHN BETON

*(tomato) (cement) (tooth) (concrete)*

Imp BEULE **EILE** SCHRITT HAST

*(dent) (hurry) (step) (haste)*

Imp **DORNEN** VERPRUEGELN ESSEN STACHELN

*(thorns) (slogging) (food) (prickles)*

Imp HALTEN DRUCKEN **STUHL** SCHEMEL

*(holding) (printing) (chair) (footstool)*

Imp REGEN **TEST** BAUCH PRUEFUNG

*(rain) (test) (belly) (exam)*

Imp **JURY** TUER SEITE GREMIUM

*(jury) (door) (page) (council)*

Imp LINIE **OBST** BETRUNKEN FRUECHTE

*(line) (fruits) (drunk) (progenies)*

Imp GABEL MANN **DUNKEL** DUESTER

*(fork) (man) (dark) (gloomy)*

Imp SALAT PFERD **ZIEHEN** REIßEN

*(salad) (horse) (pulling) (yanking)*

Imp **SCHMERZ** SERIE WAL LEIDEN

*(pain) (sequence) (whale) (woes)*

Imp KRAFT **LINIE** POST GERADE

*(power) (line) (mail) (even)*

Imp HEBEN KARTE **MASKERADE** KOSTUEM

*(lifting) (map) (masquerade) (costume)*

Imp GIFT LIGA **KRIECHEN** KRABBELN

*(poison) (league) (creeping) (crawling)*

Imp BRUSTKORB **AUTO** LADEN WAGEN

*(thorax) (car) (shop) (vehicle)*

Imp **KLAUEN** HAUSTIER GROß STEHLEN

*(stealing) (pet) (big) (thieving)*

Imp MUND FAHRRAD **OEL** FETT

*(mouth) (bicycle) (oil) (grease)*

Imp GERAEUSCH HALSBAND **WASCHEN** REINIGEN

*(sound) (collar) (washing) (cleaning)*

Imp ZAHN **KARTOFFEL** HERZ ERDAPFEL

*(tooth) (potato) (heart) (tater)*

Imp **WEINEN** FRONT SCHIFF HEULEN

*(weeping) (front) (boat) (howling)*

Imp **MUELL** BIER FARBE UNRAT

*(garbage) (beer) (color) (dross)*

Imp KUENSTLER **LUKE** WEG OEFFNUNG

*(artist) (hatchway) (path) (opening)*

Imp BABY FRUEHLING **HAUS** HUETTE

*(infant) (spring) (house) (cottage)*
